# A Unique Naphthone Derivative and a Rare 4,5-*seco*-Lanostane Triterpenoid from *Poria cocos*

**DOI:** 10.3390/molecules23102508

**Published:** 2018-09-30

**Authors:** Ting Chen, La Hua, Guixin Chou, Xudong Mao, Xianliang Zou

**Affiliations:** 1 The MOE Key Laboratory of Standardization of Chinese Medicines and SATCM Key Laboratory of New Resources and Quality Evaluation of Chinese Medicines, Institute of Chinese Materia Medica, Shanghai University of Traditional Chinese Medicine, Shanghai 201203, China; cttc1990@126.com (T.C.); qingyazizhu@163.com (L.H.); maoxd1993@163.com (X.M.); 18221816713@163.com (X.Z.); 2 Shanghai R&D Center for Standardization of Chinese Medicines, Shanghai 201203, China; 3 College of Pharmacy, Fujian University of Traditional Chinese Medicine, Fuzhou 350122, China

**Keywords:** *Poria cocos*, lanostane triterpenoids, naphthalenone derivative, cancer cell line, cytotoxic activity

## Abstract

A previously undescribed naphthalenone derivative, sohiracillinone (**1**), and a novel 4,5-*seco*-lanostane triterpenoid, 11*β*-ethoxydaedaleanic acid A (**2**) were isolated with two new lanostane triterpenoids, ceanphytamic acids A (**3**) and B (**4**), from the EtOH extract of *Poria cocos* along with 17 known compounds **5**–**21**. The absolute configuration of sohiracillinone (**1**) was unambiguously identified by NMR and electronic circular dichroism (ECD) data. The structures of other new compounds were elucidated on the basis of NMR and mass spectroscopy (MS), and the cytotoxic activities of all the isolated components were evaluated.

## 1. Introduction

*Poria cocos*, also named Fuling in Chinese, is the dried sclerotium of *P. cocos* (Schw.) Wolf, belonging to the Polyporaceae and grown in China, Japan, Korea, and North America. A well-known traditional Eastern Asian medicine, *P. cocos* extracts exert beneficial health effects and fight against illnesses [1]. *P. cocos* has important diuretic [2], antibacterial, antitumor, mitogenic, complement activating, and immune stimulating activities [3,4,5,6]. However, in the Chinese Pharmacopoeia (2015 edition), information related to the required quality standard of *P. cocos* was insufficient, and there has been little research done on *P. cocos*.

In order to determine the quality standard of *P. cocos* as well as appropriate index standards, we have studied the chemical composition of *P. cocos*. An increasing number of chemical investigations on *P. cocos* has occurred during the past decades, mainly focused on the isolation of triterpeniods and polysaccharides. Additionally, the lanostane triterpenes from *Poria cocos* (Schw.) have been reported to cause cytotoxicity towards several cancer cell lines [7,8,9]. Accordingly, we also expect to find some lead compounds which display cytotoxicity against some cancer cell lines. However, the basic skeletons of these compounds have rarely been determined and there are usually many isomers of them in *P. cocos*. In addition, the purification of lanostane triterpenes is very difficult, which makes research on their antitumor activity difficult. In a previous paper, we reported the chemical constituents of a water decoction of *P. cocos* [10]. This inspired us to conduct an additional investigation on this fungus.

**Figure 1:**
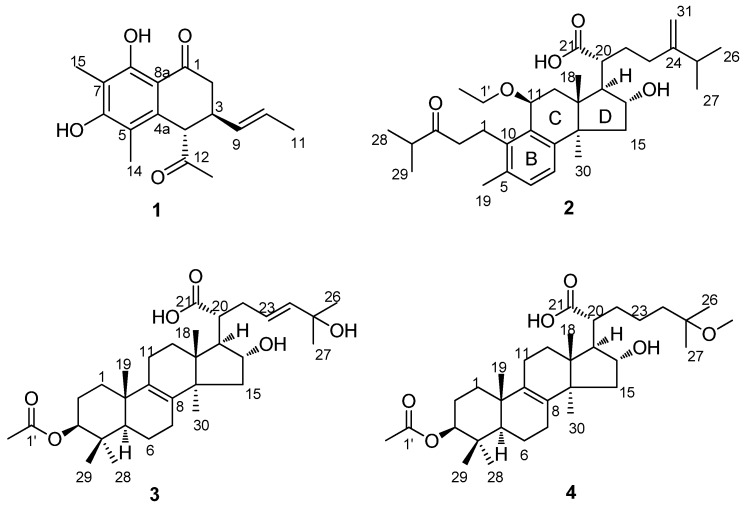
Structures of compounds **1**–**4**.

Due to the low content of lanostane triterpenes, in this paper, 100 kg dried sclerotia of *P. cocos* were extracted with 3 × 1000 L of 95% ethanol, and the chemical constituents and pharmacological characteristics of *P. cocos* were investigated. We obtained 21 compounds, including four new compounds: sohiracillinone (**1**), 11*β*-ethoxydaedaleanic acid A (**2**), ceanphytamic acid A (**3**) and ceanphytamic acid B (**4**) (Figure 1). The compounds’ cytotoxic activity was evaluated on 11 human cancer cell lines. Compound **1** showed strong cytotoxicity against the cervical cancer cell line with an IC_50_ of 12.93 ± 2.38 μM, and others showed cytotoxicity against a variety of human cancer cell lines to different degrees.

## 2. Results and Discussion

The EtOH extract of sclerotia of *P. cocos* was partitioned with petroleum ether (PE), CH_2_Cl_2_, and 1-butanol (*n*-BuOH). The CH_2_Cl_2_ layer was chromatographed repeatedly to afford four new compounds (**1**–**4** (Figure 1), along with 17 known compounds: harzianone (**5**) [11], sorbicillin (**6**) [12], 2′,3′-dihydrosorbicillin (**7**) [12], sohirnone A (**8**) [13], pachymic acid (**9**) [14], dehypachymic acid (**10**) [14], trametenolic acid (**11**) [15], deyhdrotrametenolic acid (**12**) [15], polyporenic acid C (**13**) [16], 3*β-p-*hydroxybenzoyldehydrotumulosic acid (**14**) [17], daedaleanic acid A (**15**) [18], 3*β*,15*α-*dihydroxylanosta-7,9(11),24-triene-21-oic acid (**16**) [19], poricoic acid A (**17**) [20], poricoic acid G (**18**) [21], dehydrosulphurenic acid (**19**) [22], eburicoic acid (**20**) [15], and tumulosic acid (**21**) [23]. The structures of the new compounds were established by analysis of spectroscopic data and the known ones were identified by comparison of their NMR data with those reported in the literature.

**Figure 2:**
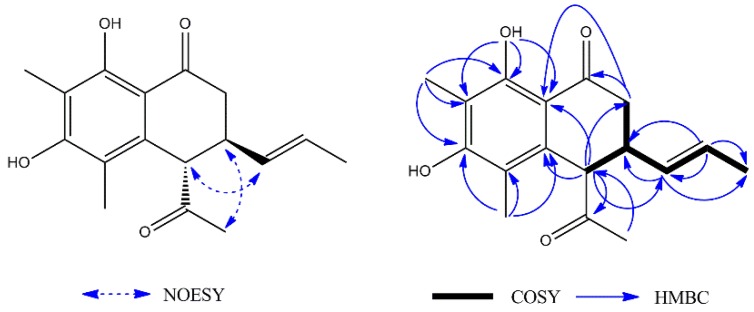
Key ^1^H–^1^H COSY, HMBC and NOESY of **1**.

**Figure 3:**
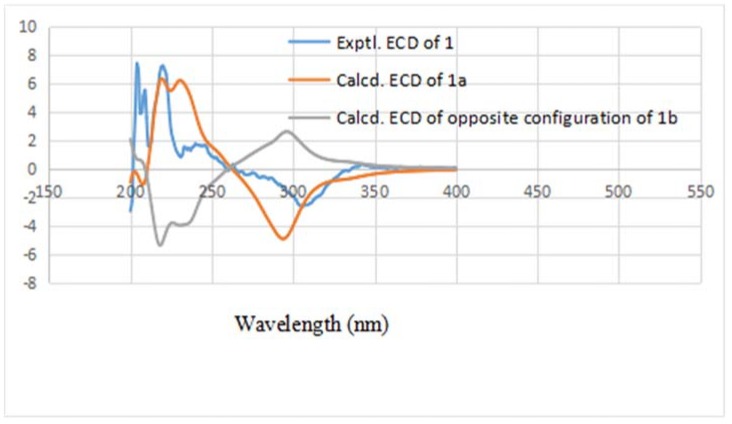
Experimental and calculated electronic circular dichroism (ECD) spectra of **1** in methanol.

Compound **1** was obtained as a yellow oil. Its high resolution electrospray ionization mass spectrum (HRESIMS) exhibited a molecular ion peak at *m/z* 288.1364, corresponding to the molecular formula of C_17_H_20_O_4_ (calcd. 288.1362), and requiring eight degrees of unsaturation. The ^13^C-NMR data showed 17 carbon signals. Analyses of the ^1^H-NMR, ^13^C-NMR (Table 1), and the HSQC spectra of **1** revealed the presence of two methyl signals in a single peak (*δ*_H_ 2.06, *δ*_C_ 11.7; *δ*_H_ 2.13, *δ*_C_ 7.5), one methyl signal in a double peak (*δ*_H_ 1.57, *J* = 6.5 Hz, *δ*_C_ 7.5), one carbonyl group (*δ*_C_ 201.6), one acetyl group (*δ*_H_ 2.24, *δ*_C_ 29.5, 207.6), and one 1,2-disubstituted double bond (*δ*_H_ 5.37, *J* = 15.0, 6.2, 0.9 Hz, *δ*_C_ 131.6; *δ*_H_ 5.52, *δ*_C_ 127.4) with a set of aromatic carbon signals for quaternary bonds (*δ*_C_ 161.6; *δ*_C_ 159.3; *δ*_C_ 136.7; *δ*_C_ 114.0; *δ*_C_ 111.5; *δ*_C_ 109.1). Additionally, the data showed a methylene group (*δ*_H_ 2.53, *J* = 17.8 Hz, *δ*_H_ 2.77, *J* = 17.8, 5.3 Hz, *δ*_C_ 39.6) and two methyne groups (*δ*_H_ 3.22, *δ*_C_ 38.6; *δ*_H_ 4.03, *δ*_C_ 55.6). These spectroscopic features together with the formula indicated that **1** was a naphthalenone derivative. The difference between **1** and naphthalenone was the substituent groups on the aromatic ring and cyclohexanone. The ^1^H–^1^H COSY spectrum (Figure 2) showed the following two fragments: –CH_2_(C-2)–CH(C-3)–CH(C-4)– and –CH(C-9)=CH(C-10)–CH_3_(C-11). In addition, in the HMBC spectrum (Figure 2), the hydrogen signal of olefin H-9 (*δ*_H_ 5.37)/H-10 (*δ*_H_ 5.52) was correlated with C-3 (*δ*_C_ 38.6), suggesting that a propenyl moiety was located at C-3. The correlations of H-13 (*δ*_H_ 2.24) with C-4 (*δ*_C_ 55.6) and H-4 (*δ*_H_ 4.03) with C-12 (*δ*_C_ 207.6) showed that an acetyl group was located at C-4. The correlations of the hydroxy group (*δ*_H_ 13.29, s, 1H) with C-7 (*δ*_C_ 109.1) and C-8 (*δ*_C_ 161.6), C-8a (*δ*_C_ 111.5) showed it was located at C-8; the correlations of H-15 (*δ*_H_ 2.13) with C-6 (*δ*_C_ 159.3), C-7 (*δ*_C_ 109.1), and C-8 (*δ*_C_ 161.6) and the correlations of H-14 (*δ*_H_ 2.06) with C-4a (*δ*_C_ 136.7), C-5 (*δ*_C_ 114.0), and C-6 (*δ*_C_ 159.3) showed that two methyl groups were situated at C-5 and C-7 on the aromatic ring. The NOESY analysis (Figure 2) showed correlations between H-3 (*δ*_H_ 3.22) and H-13 (*δ*_H_ 2.24), H-4 (*δ*_H_ 4.03) and H-9 (*δ*_H_ 5.37) which indicated the H-3 might be *α*-oriented and H-4 was *β*-oriented. According to the established relative configuration, the pair of enantiomers of **1** are (3*S*,4*S*)-**1a** and (3*R*,4*R*)-**1b**, respectively. In order to establish the absolute configuration of **1**, the electronic circular dichroism (ECD) spectrum (Figure 3) was determined, which exhibited a negative Cotton effect at 294 nm (n → π* transition). The calculated data indicated the 3*S* and 4*S* absolute configurations. Using the NMR and the CD data, the structure of **1** was determined to be (3*S*,4*S*)-4-acetyl-6,8-dihydroxy-5,7-dimethyl-3-((*E*)-prop-1-en-1-yl)-3,4-dihydronaphthalen-1(2*H*)-one, and it was named sohiracillinone.

**Figure 4:**
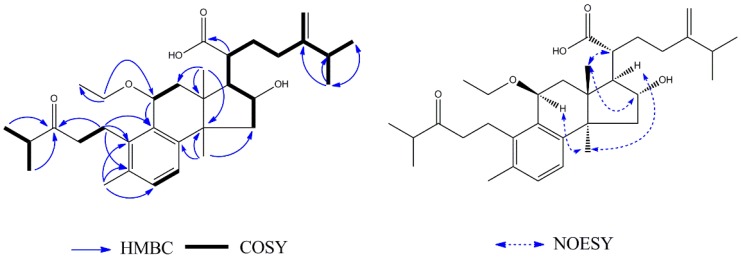
Key ^1^H–^1^H COSY, HMBC and NOESY of **2**.

Compound **2** was obtained as a white amorphous powder. Based on the HRESIMS, the molecular formula of **2** was determined to be C_33_H_50_O_5_, giving a quasi-molecular ion peak at *m/z* 525.3557 [M − H]^−^ (calcd. 525.3580). The ^1^H-NMR data (Table 2) showed signals for three methyls at *δ*_H_ 0.91 (3H, s, H_3_-18), *δ*_H_ 2.26 (3H, s, H_3_-19), *δ*_H_ 1.18 (3H, s, H_3_-30), and two mutually-coupled aromatic protons at *δ*_H_ 7.03 (1H, d, *J* = 6.2 Hz, H-6), *δ*_H_ 6.77 (1H, d, *J* = 6.2 Hz, H-7). Additionally, two pairs of equivalent secondary methyl signals were identified at *δ*_H_ 1.08 (3H, s, H_3_-28), *δ*_H_ 1.10 (3H, s, H_3_-29) and *δ*_H_ 1.05 (6H, overlap (ov.), H_3_-26 and H_3_-27). The 32 carbon signals observed in the ^13^C-NMR spectrum were sorted into eight methyls, indicating the presence of two isopropyl groups, eight methyl groups, seven methylene groups, and six methine groups, suggesting that **2** is a lanostane triterpene that is similar to daedaleanic acid A [17]. The difference between the planar structure of **2** and daedaleanic acid A is the presence of an ethoxy group at C-11. In the HMBC spectrum, H-1′ (*δ*_H_ 3.56, 1H, m; *δ*_H_ 3.38, 1H, m) was correlated with C-11 (*δ*_C_ 73.3) and CH_3_-2′ (*δ*_C_ 15.9), suggesting that the oxethyl group was located at C-11. The relative configuration of **2** was established by the NOESY experiment. Significant NOE correlations between H_3_-18 (*δ*_H_ 0.91, 3H, s) and H-16 (*δ*_H_ 4.33, 1H, t) and H-20 (*δ*_H_ 2.57, 1H, m), and between H_3_-30 (*δ*_H_ 1.18, 3H, s) and H-17 (*δ*_H_ 2.30, 1H, m) and H-11 (*δ*_H_ 4.47, 1H, d) indicated that OH-16 and H-17 have *α*-orientations. From the analysis of all of these data and from a biosynthetic point of view, the structure of **2** was assigned as shown in Figure 4 and named 11*β*-ethoxydaedaleanic acid A.

**Figure 5:**
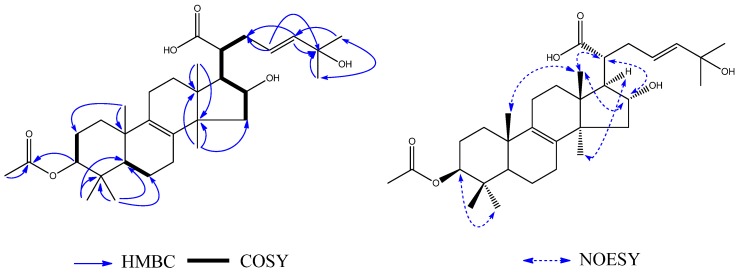
Key ^1^H–^1^H COSY, HMBC and NOESY of **3**.

Compound **3** was obtained as a white amorphous powder. The HRESIMS showed a *m/z* at 530.3616 calcd. for C_32_H_49_O_6_, 530.3607 [M − H]^−^ indicated a molecular formula of C_32_H_50_O_6_. Its ^1^H-NMR (Table 2) spectrum showed the signals of eight methyl signals at *δ*_H_ 0.79 (3H, s, H_3_-18), *δ*_H_ 1.02 (3H, s, H_3_-19), *δ*_H_ 1.24 (3H, s, H_3_-26), *δ*_H_ 1.24 (3H, s, H_3_-27), *δ*_H_ 0.89 (3H, s, H_3_-28), *δ*_H_ 0.91 (3H, s, H_3_-29), *δ*_H_ 1.13 (3H, s, H_3_-30) and *δ*_H_ 2.03 (3H, s, H_3_-2′) and two oxygen-bearing methynes at *δ*_H_ 4.45 (1H, t, H-3) and *δ*_H_ 4.05 (1H, t, H-16). Its ^13^C-NMR (Table 2) and HSQC spectra proved the existence of eight methyl groups, as well as an acetyl carbon at *δ* 172.9 (C, C-1′) and a pair of tetrasubstituted olefinic carbonds at *δ* 135.6 (C, C-8) and *δ* 136.0 (C, C-9) and three oxygenated carbons at *δ* 82.5 (CH, C-3), *δ* 77.9 (CH, C-16), *δ* 71.1 (C, C-25). In addition, the ^13^C NMR and HSQC spectra also exhibited two olefinic signals at *δ*_C_ 126.4 (*δ*_H_ 5.62, 1H, ov., C-23) and *δ*_C_ 140.2 (*δ*_H_ 5.62, 1H, ov., C-24). There were ^1^H–^1^H COSY (Figure 5) correlations between H-2 (*δ*_H_ 1.66) and H-3 (*δ*_H_ 4.45), between H-5 (*δ*_H_ 1.16) and H-6 (*δ*_H_ 1.66), between H-15 (*δ*_H_ 2.18), H-16 (*δ*_H_ 4.05), H-17 (*δ*_H_ 2.06), H-20 (*δ*_H_ 2.32), H-22 (*δ*_H_ 2.50), and H-23 (*δ*_H_ 5.62). Therefore, it can be concluded that **3** was a lanost-8-ene triterpenoid. On the basis of the HMBC (Figure 5), correlations from H_3_-26 (*δ*_H_ 1.24) to CH_3_-27 (*δ*_C_ 29.8), H_3_-27 (*δ*_H_ 1.24) to CH_3_-26 (*δ*_C_ 29.8), H_3_-27 (*δ*_H_ 1.24) to C-25 (*δ*_C_ 71.1), and H_3_-26 (*δ*_H_ 1.24) to C-24 (*δ*_C_ 140.2) indicated the presence of a hydroxy group at C-25. The correlation from H-3 (*δ*_H_ 4.45) to C-1′ (*δ*_C_ 172.9) showed that the acetyl group was located at C-3. The NOE correlations between H-16 (*δ*_H_ 4.05) and H-20 (*δ*_H_ 2.32), and between H_3_-30 (*δ*_H_ 1.13) and H-17 (*δ*_H_ 2.06) indicated *α*-orientations for OH-16 and H-17. The correlation between H_3_-18 (*δ*_H_ 0.79) and H_3_-19 (*δ*_H_ 1.02), H-16 (*δ*_H_ 4.05) and H-20 (*δ*_H_ 2.32) showed that CH_3_-18, CH_3_-19 and H-20 have *β*-orientations. Thus, the structure of **3** was determined to be 3*β*-acetoxy-16*α*,25-dihydroxylanost-8,23-dien-21-oic acid, and it was named ceanphytamic acid A (Figure 5).

**Figure 6:**
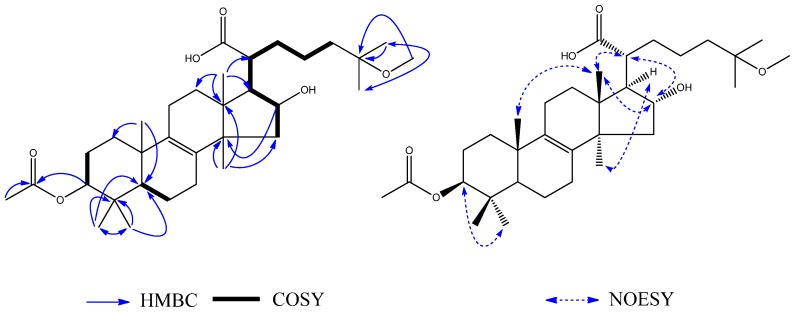
Key ^1^H–^1^H COSY, HMBC and NOESY of **4**.

Compound **4** was obtained as a white amorphous powder. The HRESIMS showed a *m/z* at 546.3913 calcd. for C_33_H_54_O_6_, 546.3920 [M + H]^+^. ^1^H–^1^H COSY (Figure 6) correlations were shown between H-2 (*δ*_H_ 1.72) and H-3 (*δ*_H_ 4.59), between H-5 (*δ*_H_ 1.04) and H-6 (*δ*_H_ 1.61), and between H-15 (*δ*_H_ 2.34), H-16 (*δ*_H_ 4.47), H-17 (*δ*_H_ 2.30), H-20 (*δ*_H_ 2.86), H-22 (*δ*_H_ 1.24), H-23 (*δ*_H_ 2.33), and H-24 (*δ*_H_ 1.41). The ^1^H-NMR showed nine methyl groups at H_3_-18 (*δ*_H_ 1.05, 3H, s), H_3_-19 (*δ*_H_ 0.89, 3H, s), H_3_-26 (*δ*_H_ 1.01, 3H, s), H_3_-27 (*δ*_H_ 1.00, 3H, s), H_3_-28 (*δ*_H_ 0.84, 3H, s), H_3_-29 (*δ*_H_ 0.86, 3H, s), H_3_-30 (*δ*_H_ 1.40, 3H, s), H_3_-2′ (*δ*_H_ 2.02, 3H, s), and H_3_-OMe (*δ*_H_ 3.08, 3H, s). The ^13^C-NMR (Table 2) and HSQC spectrum proved the existence of nine methyl groups, as well as an acetyl carbon at C-1′ (*δ*_C_ 170.8) and three oxygenated carbons at C-3 (*δ*_C_ 80.7), C-16 (*δ*_C_ 76.6), and C-25 (*δ*_C_ 74.4). The signals observed in the ^1^H NMR and ^13^C NMR spectra (Table 2) closely resembled those of **3** except for the partial signals of the C-17 side-chain and the presence of an additional methyl group **3**, indicating that **4** also had the same lanostane skeleton as **3**. On the basis of the HMBC (Figure 6) correlations between H_3_-26 (*δ*_H_ 1.01) and CH_3_-27 (*δ*_C_ 25.0), H_3_-27 (*δ*_H_ 1.00) and CH_3_-26 (*δ*_C_ 25.0), H_3_-26 (*δ*_H_ 1.01) and C-25 (*δ*_C_ 74.4), and H_3_-OMe (δ_H_ 3.08) and CH_3_-25 (*δ*_C_ 74.4), the presence of a methoxy group at C-25 was indicated. The NOE correlations between H_3_-30 (*δ*_H_ 1.40) and H-17 (*δ*_H_ 2.30) indicated *α*-orientations for H-17. The correlations between H_3_-18 (*δ*_H_ 1.05) and H_3_-19 (*δ*_H_ 0.89), and between H-16 (*δ*_H_ 4.47) and H-20 (*δ*_H_ 2.86) indicated *β*-orientations for CH_3_-18, CH_3_-19 and H-20. Therefore, the structure of **4** (Figure 6) was determined to be 3*β*-acetoxy-16*α*-hydroxylanost-25-methoxyl-8-en-21-oic acid, and it was named ceanphytamic acid B.

All of the isolated compounds were evaluated for cytotoxicity against eleven human cancer cell lines: A549 (adenocarcinomic human alveolar basal epithelial cells, ATCC: CCL-185), Calu-1 (human lung cancer cells, ATCC: HTB-54), HeLa (human cervical cancer cells, ATCC: CCL-2), MDA-MB-231 (human breast invasive ductal carcinoma cells, ATCC: HTB-26), SW579 (human thyroid squamous cell carcinoma cells, ATCC: HTB-107), SK-OV-3 (human ovarian cancer cells, ATCC: HTB-77), T84 (human lung cancer cells, ATCC: CCL-248), Hep G2 (human liver cancer cells, ATCC: HB-8065), Hep 3B2.1-7 (human liver cancer cells, ATCC: HB-8064), KATO III (human gastric carcinoma cells, ATCC: HTB-103), MCF-7 (human breast adenocarcinoma cell, ATCC: HTB-22) by the CCK-8 method. None of compounds exhibited cytotoxicity against T84 cells, Hep G2 cells, Hep 3B2.1-7 cells, KATO III cells, or MCF-7 cells. Compound **1** showed stronger cytotoxicity against HeLa cells than the other tested compounds, and **2**–**4** showed different degree cytotoxicity against A549, SK-OV-3 and SW579 cells (Table 3). Compounds **5**–**6**, **9**–**10** and **12**–**14** showed selective inactive cytotoxicity against these cancer cell lines. However, the cytotoxic activities were far less potent compared to those of the positive controls.

## 3. Experimental Section

### 3.1. General Information

Optical rotations were obtained with an Autopol VI polarimeter (No. 90079, Rudolph Research Analytical, Hackettstown, NewJersey, USA). IR spectra were recorded with a Perkin Elmer FT-IR spectrometer (Beijing Purkinje General Istrument Co., Ltd., Beijing, People’s Republic of China). One-dimensional and two-dimensional NMR spectra were run on a AVANCE-III instrument (Bruker, Bremen, Germany) operating at 600/400 MHz for ^1^H, 125/100 MHz for ^13^C, respectively, with tetramethylsilane as an internal standard. HRESIMS spectra were obtained on a UPLC Premier QTOF spectrometer (Waters, Milford, Massachusetts, USA). Reversed phase medium pressure liquid chromatography (RP-MPLC) was performed on a GRACE system (Grace, Columbia, MD, USA). Preparative HPLC was performed on an 1260 series instrument (Agilent, Santa Clara, CA, USA) equipped with a Capcellpak C18 column (250 × 20 mm, 5 μm, flow rate: 16 mL/min, Shiseido, Tokyo, Japan). Column chromatography were performed with silica gel (200–300 mesh, Qingdao Haiyang Chemical Co., Ltd., Qingdao, China), Sephadex LH-20 (GE Healthcare Bio-Sciences AB, Stockholm, Sweden), and YMC gel ODS-AQ (50 µm, YMC Co., Ltd., Kyoto, Japan). Thin-layer chromatography (TLC) and preparative TLC were carried out on HSGF254 plates (Yantai Jiangyou Silica Gel Development Co., Ltd., Yantai, China).

### 3.2. Plant Material

The sclerotia of *Poria cocos* was purchased from Shanghai Kang Qiao Herbal Pieces Co., Ltd. (Shanghai, China), and identified by Professor Hong Xu from Shanghai University of Traditional Chinese Medicine. A voucher specimen (150910) was deposited at Shanghai R&D Center for standardization of Chinese Medicines, Shanghai, China.

### 3.3. Extraction and Isolation

Dried sclerotia of *P. cocos* (100 kg) was extracted three times, each time with 95% EtOH (1000 L) to give a crude extract (2300 g), which was suspended in water and successively partitioned with petroleum ether (PE), CH_2_Cl_2_ and *n*-BuOH. The CH_2_Cl_2_ extracts (804 g) were loaded into silica gel column chromatography (200–300 mesh) and eluted with a stepwise gradient of PE-EtOAc (100:1 to 1:1) and EtOAc-MeOH (20:1 to 1:1) to obtain 13 fractions (Fr. A-Fr. M) on the basis of their TLC characteristics.

Fraction B (18.4 g) was repeatedly chromatographed on silica gel (PE/EtOAc = 50:1 to 1:1) and Sephadex LH-20 (MeOH) columns to obtain six fractions (Fr. B-1 to Fr. B-6). Fr. B-2 (1.7 g) was subjected to an ODS preparative MPLC column eluted with MeOH-H_2_O (3:7 to 8:2, *v/v*), followed by Sephadex LH-20 (MeOH) and preparative HPLC (86% CH_3_CN in H_2_O, 16 mL/min) to give harzianone (**5**, 2.1 mg). Fr. B-3 (2.2 g) was chromatographed by preparative HPLC (82% CH_3_CN in H_2_O, 16 mL/min) to yield sorbicillin (**6**, 4.5 mg), 2′,3′-dihydrosorbicillin (**7**, 3.7 mg), sohirnone A (**8**, 6.2 mg). Fr. B-5 (4.0 g) was eluted repeatedly with MeOH-H_2_O (1:7 to 9:1, *v/v*) and preparative HPLC to yield sohiracillinone (**1**, 1.9 mg), 11*β*-ethoxydaedaleanic acid A (**2**, 1.6 mg), and daedaleanic acid A (**15**, 2.0 mg), respectively.

Fr. C (21.0 g) was repeatedly chromatographed on silica gel (PE/EtOAc = 50:1 to 1:1) to obtain seven fractions (Fr. C-1 to Fr. C-7). Fr. C-1 (11.5 g) was subjected to an ODS preparative MPLC column eluted with MeOH-H_2_O (4:5 to 9:1, *v/v*), and followed by preparative HPLC to yield pachymic acid (**9**, 0.6 g), dehypachymic acid (**10**, 0.5 g), and polyporenic acid C (**13**, 0.3 g), respectively. Fr. C-2 was also isolated by the same method as Fr.C-1 to yield deyhdrotrametenolic acid (**12**, 4.5 mg), trametenolic acid (**11**, 3.2 mg), eburicoic acid (**20**, 3.7 mg), and 3*β-p-*hydroxybenzoyl-dehydrotumulosic acid (**14**, 2.8 mg), respectively.

Fr. D (9.5 g) was repeatedly chromatographed on silica gel (PE/EtOAc = 50:1 to 1:1) to obtain five fractions (Fr. D-1 to Fr. D-5). Fr. D-2 (1.5 g) was subjected to an ODS preparative MPLC column eluted with MeOH-H_2_O (1:5 to 9:1, *v/v*), and followed by preparative HPLC (84% CH_3_CN in H_2_O, 16 mL/min) to yield ceanphytamic acid A (**3**, 2.1 mg). Fr. D-4 (2.2 g) was subjected to an ODS preparative MPLC column eluted with MeOH-H_2_O (1:8 to 9:1, *v/v*), and followed by preparative HPLC (82% CH_3_CN in H_2_O, 16 mL/min) to yield poricoic acid A (**17**, 3.1 mg), and poricoic acid G (**18**, 3.7 mg).

Fr. F (12.7 g) was repeatedly chromatographed on silica gel (PE/EtOAc = 50:1 to 1:1) to obtain seven fractions (Fr. F-1 to Fr. F-7). Fr. F-3 (0.3 g) was eluted by HPLC (84% CH_3_CN in H_2_O, 16 mL/min) to yield ceanphytamic acid B (**4**, 2.0 mg). Fr. F-5 (0.8 g) was eluted by HPLC (84% CH_3_CN in H_2_O, 16 mL/min) to yield 3*β*,15*α-*dihtdroxylanosta-7,9(11),24-trien-21-oic acid (**16**, 3.1 mg).

Fr. G (7.1 g) was repeatedly chromatographed on silica gel (PE/EtOAc = 50:1 to 1:1) to obtain three fractions (Fr. G-1 to Fr. G-3). Fr. G-1 was eluted by HPLC (78% CH_3_CN in H_2_O, 16 mL/min) to yield dehydrosulphurenic acid (**19**, 3.8 mg).

Fr. H (5.1 g) was repeatedly chromatographed on silica gel (PE/EtOAc = 100:1 to 1:1) to obtain three fractions (Fr. H-1 to Fr. H-3). Fr. H-2 was subjected to an ODS preparative MPLC column eluted with MeOH-H_2_O (1:9 to 9:1, *v/v*) to yield tumulosic acid (**21**, 3.9 mg).

### 3.4. Spectroscopic Data

*Sohiracillinones* (**1**): Yellow oil; ${\left[\alpha \right]}_{D}^{20}$ = −19.2 (*c* = 0.033, CHCl_3_). IR: 2920, 2852, 1740, 1596, 1413, 1366, 1099, 779, 720, 616 cm^−1^. ^1^H and ^13^C NMR are shown in Table 1. Negative HRESIMS: 288.1364 ([M − H]^−^, C_17_H_19_O_4_; calcd. 288.1362). More Spectroscropic data can be found in the Supplementary Materials (Figures S1–S35).

*11β-Ethoxydaedaleanic acid A* (**2**): White amorphous powder; ${\left[\alpha \right]}_{D}^{20}$ = +6.7 (*c* = 0.1, C_5_H_5_N). IR: 3395, 2919, 2849, 1645, 1438, 1069, 804, 760, 703 cm^−1^. ^1^H and ^13^C NMR are shown in Table 2. Negative HRESIMS: 525.3557 ([M − H]^−^, C_33_H_49_O_5_; calcd. 525.3580). More Spectroscropic data can be found in the Supplementary Materials (Figures S1–S35).

*Ceanphytamic acid A* (**3**): White amorphous powder; ${\left[\alpha \right]}_{D}^{20}$ = +16.7 (*c* = 0.03, C_5_H_5_N). IR: 3676, 2988, 2902, 1702, 1635, 1394, 1075, 1066, 1056, 892 cm^−1^. ^1^H and ^13^C NMR are shown in Table 2. Negative HRESIMS: 530.3616 ([M − H]^−^, C_32_H_49_O_6_; calcd. 530.3607). More Spectroscropic data can be found in the Supplementary Materials (Figures S1–S35).

*Ceanphytamic acid B* (**4**): White amorphous powder; ${\left[\alpha \right]}_{D}^{20}$ = +19.4 (*c =* 0.03, C_5_H_5_N). IR: 3676, 2988, 2902, 1707, 1620, 1407, 1394, 1251, 1066, 1057, 891 cm^−1^. ^1^H and ^13^C NMR are shown in Table 2. Positive HRESIMS: 546.3913 ([M + H]^+^, C_33_H_53_O_6_; calcd. 546.3920). More Spectroscropic data can be found in the Supplementary Materials (Figures S1–S35).

### 3.5. Cell Culture

Nine human cancer cell lines, A549, Calu-1, HeLa, SK-OV-3, T84, Hep G2, Hep 3B2.1-7, KATO III, and MCF-7, were kindly provided by Stem Cell Bank, Chinese Academy of Sciences. Two human cancer cell lines, MDA-MB-231 and SW579, were kindly provided by Professor Hong Xu from Shanghai University of Traditional Chinese Medicine. A Cell Counting Kit-8 was purchased from Shanghai DOJINDO Co., Ltd. (Shanghai, China). Hep G2, Hep 3B2.1-7, HeLa, and MCF-7 were maintained in Dulbecco’s modified Eagle’s medium (DMEM) supplemented with 10% (*v/v*) heat-inactivated fetal bovine serum (FBS) and 0.1% (*v/v*) penicillin-streptomycin in a humidified incubator with 5% CO_2_ at 37 °C. Calu-1 and SK-OV-3 were maintained in McCoy’s 5A supplemented with 10% (*v/v*) heat-inactivated FBS and 0.1% (*v/v*) penicillin-streptomycin in a humidified incubator with 5% CO_2_ at 37 °C. A549 was maintained in F12K supplemented with 10% (*v/v*) heat-inactivated FBS and 0.1% (*v/v*) penicillin-streptomycin in a humidified incubator with 5% CO_2_ at 37 °C. KATO III, MDA-MB-231, and SW579 were maintained in RPMI 1640 supplemented with 10% (*v/v*) heat-inactivated FBS and 0.1% (*v/v*) penicillin-streptomycin in a humidified incubator with 5% CO_2_ at 37 °C. T84 was maintained in D-MEM/F-12 supplemented with 20% (*v/v*) heat-inactivated FBS and 0.1% (*v/v*) penicillin-streptomycin in a humidified incubator with 5% CO_2_ at 37 °C. All cell culture media and reagents were purchased from Thermo Scientific Hyclone (Logan, UT, USA).

### 3.6. Cell Assay

The compounds’ cytotoxicity was measured by the CCK-8 method. Compounds were dissolved with dimethylsulfoxide (DMSO). The cells were seeded onto 96-well microplates at a density of 1 × 10^4^ cells per well in 100 μL of medium each. After incubation at 37 °C in a humidified incubator for 24 h, the cells were treated with various concentrations of each compound. After incubation, 10% CCK-8 (10 μL in 90 μL FBS) was added to each well of the plate. After removal from the medium, the cells were incubated at 37 °C for 1 h. Cell viability was calculated as a percentage of viable cells in the compound-treated group vs. the control group by the following equation: Cell viability (%) = [1 − OD (Compound)/OD (Blank)] × 100%.

## 4. Conclusions

In sum, one new naphthone derivative, sohiracillinone (**1**), and three new triterpenoids, 11*β*-ethoxydaedaleanic acid A (**2**), ceanphytamic acid A (**3**), and ceanphytamic acid B (**4**), were characterized from the EtOH extract of the sclerotia of *Poria cocos*. The skeleton of **1** was unique and the absolute configurations was determined by ECD. Moreover, a rare 4,5-*seco*-lanostane triterpenoid **2** and two unreported side-chain of lanostane triterpenoids **3** and **4** were also isolated. This adds new facets to the structural diversity of the *P. cocos* family. Our findings showed that **1** has stronger cytotoxicity against HeLa cells than the other tested compounds, and the other compounds showed selective inactive cytotoxicity against the testing cancer cell lines. Even though the level of cytotoxic activity was not significant, but these findings also suggest that *P. cocos* may have the potential to be a useful therapeutic natural source for cancer prevention. Besides, following the study of the compounds’ chemistry, we propose that lanostane triterpenoids is a reasonable index standard for *P. cocos*.

## Figures and Tables

**Table 1:** ^1^H- and ^13^C-NMR Data for **1** in CDCl_3_.

Position	*δ*_C_^a^	*δ*_H_^b^ (*J* in Hz)
1	201.6, C	-
2	39.6, CH_2_	2.53, br. d (17.8) 2.77, dd (17.8, 5.3)
3	38.6, CH	3.22, s
4	55.6, CH	4.03, br. s
4a	136.7, C	-
5	114.0, C	-
6	159.3, C	-
7	109.1, C	-
8	161.6, C	-
8a	111.5, C	-
9	131.6, CH	5.37, ddd (15.0, 6.2, 0.9)
10	127.4, CH	5.52, m
11	18.1, CH_2_	1.57, d (6.2)
12	207.6, C	-
13	29.5, CH_3_	2.24, s
14	11.7, CH_3_	2.06, s
15	7.5, CH_3_	2.13, s
8-OH	-	13.29, s

^a^ Recorded at 125 MHz; ^b^ Recorded at 400 MHz.

**Table 2:** ^1^H- and ^13^C-NMR data for compounds **2**–**4**.

No.	Compound 2 *		Compound 3 **		Compound 4 ***	
	*δ*_C_^a^	*δ*_H_^b^ (*J* in Hz)	*δ*_C_^a^	*δ*_H_^b^ (*J* in Hz)	*δ*_C_^a^	*δ*_H_^b^ (*J* in Hz)
1	24.5, CH_2_	2.85, ov. 2.72, ov.	36.4, CH_2_	1.78, ov. 1.27, ov.	35.3, CH_2_	1.53, ov. 1.09, ov.
2	40.6, CH_2_	2.83, ov. 2.68, ov.	25.1, CH_2_	1.66, ov.	24.4, CH_2_	1.72, m 1.62, m
3	214.9, C	-	82.5, CH	4.45, t (9.2, 7.0)	80.7, CH	4.59, dd (7.6, 2.8)
4	41.1, CH	2.56, m	38.9, C	-	37.9, C	-
5	134.4, C	-	52.0, CH	1.16, ov.	50.7, CH	1.04, ov.
6	130.4, CH	7.03, d (6.2)	19.2, CH_2_	1.65, ov. 1.76, ov.	18.3, CH_2_	1.61, m 1.39, ov.
7	122.8, CH	6.77, d (6.2)	27.6, CH_2_	2.08, ov.	26.6, CH_2_	1.97, ov. 2.00, ov.
8	144.9, C	-	135.6, C	-	134.9, C	-
9	133.7, C	-	136.0, C	-	134.4, C	-
10	140.9, C	-	38.2, C	-	37.0, C	-
11	73.3, CH	4.47, d (4.2)	21.2, CH_2_	2.07, ov. 2.99, ov.	20.8, CH_2_	1.82, ov. 2.01, ov.
12	33.9, CH_2_	2.28, ov.	30.1, CH_2_	1.59, m 1.82, m	29.7, CH_2_	2.15, m 1.82, ov.
13	44.2, C	-	47.0, C	-	46.1, C	-
14	49.8, C	-	49.4, C	-	48.6, C	-
15	44.1, CH_2_	1.81, d (10.6)	43.6, CH_2_	2.18, m 1.26, m	43.4, CH_2_	2.34, m 1.65, m
16	77.6, CH	4.33, t (5.9)	77.9, CH	4.05, t (7.8, 6.6)	76.6, CH	4.47, m
17	56.7, CH	2.30, m	57.3, CH	2.06, m	57.2, CH	2.30, m
18	19.5, CH_3_	0.91, s	17.8, CH_3_	0.79, s	17.7, CH_3_	1.05, s
19	19.8, CH_3_	2.26, s	19.6, CH_3_	1.02, s	19.1, CH_3_	0.89, s
20	46.4, CH	2.57, m	51.6, CH	2.32, m	48.7, CH	2.86, m
21	- ^c^, C	-	- ^c^, C	-	179.1, C	-
22	30.6, CH_2_	1.99, m 1.18, m	36.4, CH_2_	2.50, d (12.7) 2.26, m	31.3, CH_2_	1.24, t (4.8) 1.18, ov.
23	32.4, CH_2_	2.03, m	126.4, CH	5.62, ov.	33.3, CH_2_	2.33, m 2.24, m
24	155.1, C	-	140.2, CH	5.62, ov.	30.3, CH_2_	1.41, m 1.47, m
25	33.3, CH	2.08, m	71.1, C	-	74.4, C	-
26	22.1, CH_3_	1.05, s	29.8, CH_3_	1.24, s	25.0, CH_3_	1.01, s
27	21.9, CH_3_	1.05, s	29.8, CH_3_	1.24, s	25.0, CH_3_	1.00, s
28	18.4, CH_3_	1.08, s	28.5, CH_3_	0.89, s	27.9, CH_3_	0.84, s
29	18.6, CH_3_	1.10, s	16.9, CH_3_	0.91, s	16.7, CH_3_	0.86, s
30	30.5, CH_3_	1.18, s	25.7, CH_3_	1.13, s	25.3, CH_3_	1.40, s
31	107.3, CH_2_	4.77, d (18.4)	-	-	-	-
1′	63.0	3.56, m 3.38, m	172.9, C	-	170.8, C	-
2′	15.9	1.08, s	21.1, CH_3_	2.03, s	21.1, CH_3_	2.02, s
O-Me	-	-	-	-	49.0, CH_3_	3.08, s

^a^ Recorded at 125 MHz; ^b^ recorded at 400 MHz; **^c^** unable to be measured; * dissolution by CDCl_3_; ** dissolution by CD_3_OD; *** dissolution by C_5_D_5_N-*d*_5_.

**Table 3:** Cytotoxic activity of **1**–**4** against human cancer cell lines (IC_50_ [μM] ^a^) ^b^.

Comp.	Cell Lines (IC_50_) μM				
	A549	HeLa	MDA-MB-231	SK-OV-3	SW579
1	45.18 ± 5.11	12.93 ± 2.38	81.68 ± 3.99	˃100	˃100
2	61.05 ± 2.51	˃100	˃100	81.13 ± 10.76	˃100
3	˃100	54.41 ± 7.01	˃100	72.38 ± 8.77	69.52 ± 8.19
4	˃100	˃100	˃100	70.01 ± 2.29	81.62 ± 6.26

^a^ All data were represented the mean ± SD of triplicate experiments. ^b^ 5FU (fluorouracil), DXR (doxorubicin), CDDP (cisplatin), and PTX (paclitaxel) were used as the positive controls.

## Supplementary Materials

The following are available online: Figures S1–S35.
