# Supplementary material for: A Unique Naphthone Derivative and a Rare 4,5-seco-Lanostane Triterpenoid from Poria cocos
Source: Molecules. 2018 Sep 30;23(10):2508. doi: 10.3390/molecules23102508 (PMC6222825; doi:10.3390/molecules23102508)
Supplement: Supplementary file 1 [file molecules-23-02508-s001.pdf]

Supporting Information for

A unique naphthone derivative and a rare 4,5-*seco*-lanostane triterpenoid from *Poria cocos*

Ting Chen [1,2,3], La Hua [1,2], Guixin Chou [1,2], Xudong Mao [1,2], Xianliang Zou [1,2]

Affiliation

[1] The MOE Key Laboratory of Standardization of Chinese Medicines, and SATCM Key Laboratory of New Resources and Quality Evaluation of Chinese Medicines, Institute of Chinese Materia Medica, Shanghai University of Traditional Chinese Medicine, Shanghai 201203, People's Republic of China

[2] Shanghai R&D Center for Standardization of Chinese Medicines, Shanghai 201203, People's Republic of China

[3] College of Pharmacy, Fujian University of Traditional Chinese Medicine, Fuzhou, Fujian 350122, People's Republic of China

Correspondence

Prof. Dr. Guixin Chou. The MOE Key Laboratory of Standardization of Chinese Medicines, and SATCM Key Laboratory of New Resources and Quality Evaluation of Chinese Medicines, Institute of Chinese Materia Medica, Shanghai University of Traditional Chinese Medicine, Shanghai 201203, People's Republic of China. Shanghai R&D Center for Standardization of Chinese Medicines, Shanghai 201203, People's Republic of China. E-mail : chouguixinzyb@126.com (G.-X. Chou)

Tel: +86-21-50271706. Fax: +86-21-50271708.

Figure S1. ^{1}H NMR spectrum of sohiracillinone (**1**) in $CDCl_3$

Figure S2. ^{13}C NMR spectra of sohiracillinone (**1**) in $CDCl_3$

Figure S3. HSQC spectrum of sohiracillinone (**1**) in $CDCl_3$

Figure S4. HMBC spectrum of sohiracillinone (**1**) in $CDCl_3$

Figure S5. ^{1}H-^{1}H COSY spectrum of sohiracillinone (**1**) in $CDCl_3$

Figure S6. NOESY spectrum of sohiracillinone (**1**) in $CDCl_3$

Figure S7. IR spectrum of sohiracillinone (**1**)

Figure S8. HRESIMS spectrum of sohiracillinone (**1**)

Figure S9. ^{1}H NMR spectrum of 11*β*-ethoxy-daedaleanic acid A (**2**) in $CDCl_3$

Figure S10. ^{13}C NMR spectra of 11*β*-ethoxy-daedaleanic acid A (**2**) in $CDCl_3$

Figure S11. HSQC spectrum of 11*β*-ethoxy-daedaleanic acid A (**2**) in $CDCl_3$

Figure S12. HMBC spectrum of 11*β*-ethoxy-daedaleanic acid A (**2**) in $CDCl_3$

Figure S13. ^{1}H-^{1}H COSY spectrum of 11*β*-ethoxy-daedaleanic acid A (**2**) in $CDCl_3$

Figure S14. NOESY spectrum of 11*β*-ethoxy-daedaleanic acid A (**2**) in $CDCl_3$

Figure S15. Dept spectrum of 11*β*-ethoxy-daedaleanic acid A (**2**) in $CDCl_3$

Figure S16. IR spectrum of 11*β*-ethoxy-daedaleanic acid A (**2**)

Figure S17. HRESIMS spectrum of 11*β*-ethoxy-daedaleanic acid A (**2**)

Figure S18. ^{1}H NMR spectrum of ceanphytamic acid A (**3**) in CD_3OD

Figure S19. ^{13}C NMR spectra of ceanphytamic acid A (**3**) in CD_3OD

Figure S20. HSQC spectrum of ceanphytamic acid A (**3**) in CD_3OD

Figure S21. HMBC spectrum of ceanphytamic acid A (**3**) in CD_3OD

Figure S22. ^{1}H-^{1}H COSY spectrum of ceanphytamic acid A (**3**) in CD_3OD

Figure S23. NOESY spectrum of ceanphytamic acid A (**3**) in CD_3OD

Figure S24. Dept spectrum of ceanphytamic acid A (**3**) in CD_3OD

Figure S25. IR spectrum of ceanphytamic acid A (**3**)

Figure S26. HRESIMS spectrum of ceanphytamic acid A (**3**)

Figure S27. ^{1}H NMR spectrum of ceanphytamic acid B (**4**) in C_5D_5N-*d5*

Figure S28. ^{13}C NMR spectra of ceanphytamic acid B (**4**) in C_5D_5N-*d5*

Figure S29. HSQC spectrum of ceanphytamic acid B (**4**) in C_5D_5N-*d5*

Figure S30. HMBC spectrum of ceanphytamic acid B (**4**) in C_5D_5N-*d5*

Figure S31. ^{1}H-^{1}H COSY spectrum of ceanphytamic acid B (**4**) in C_5D_5N-*d5*

Figure S32. NOESY spectrum of ceanphytamic acid B (**4**) in C_5D_5N-*d5*

Figure S33. Dept spectrum of ceanphytamic acid B (**4**) in C_5D_5N-*d5*

Figure S34. IR spectrum of ceanphytamic acid B (**4**)

Figure S35. HRESIMS spectrum of ceanphytamic acid B (**4**)

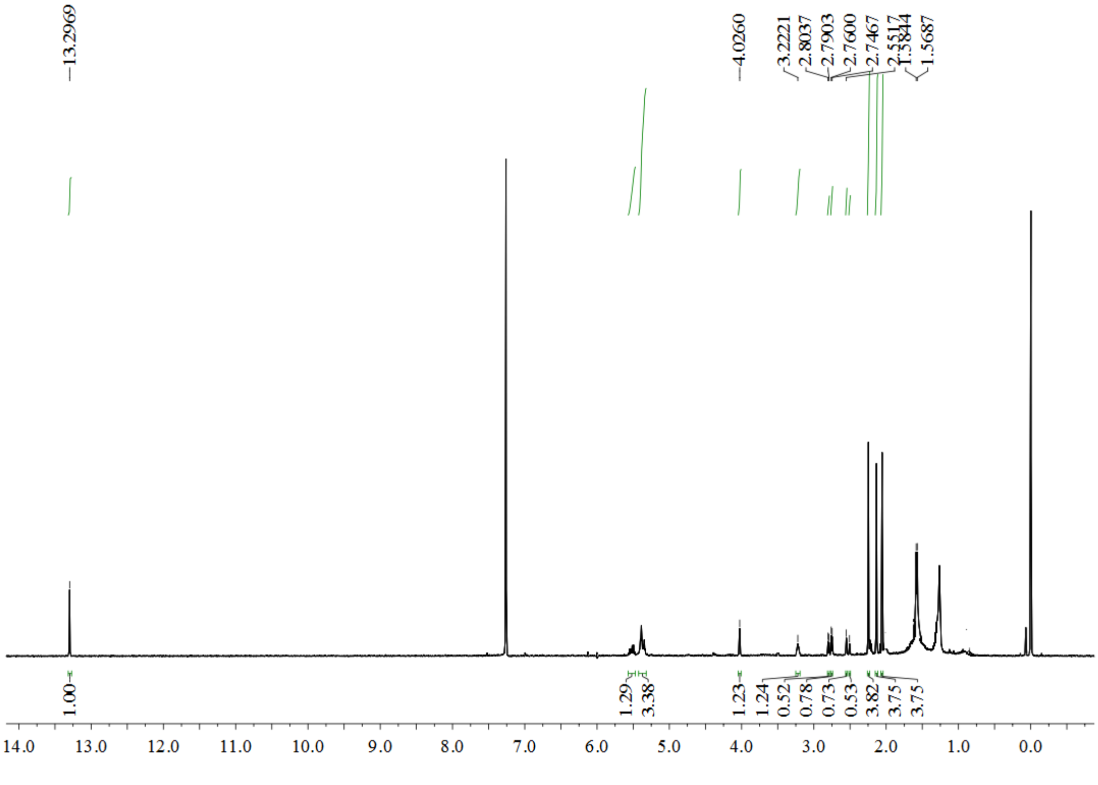

Figure S1. ^{1}H NMR spectrum of sohiracillinone (**1**) in $CDCl_3$

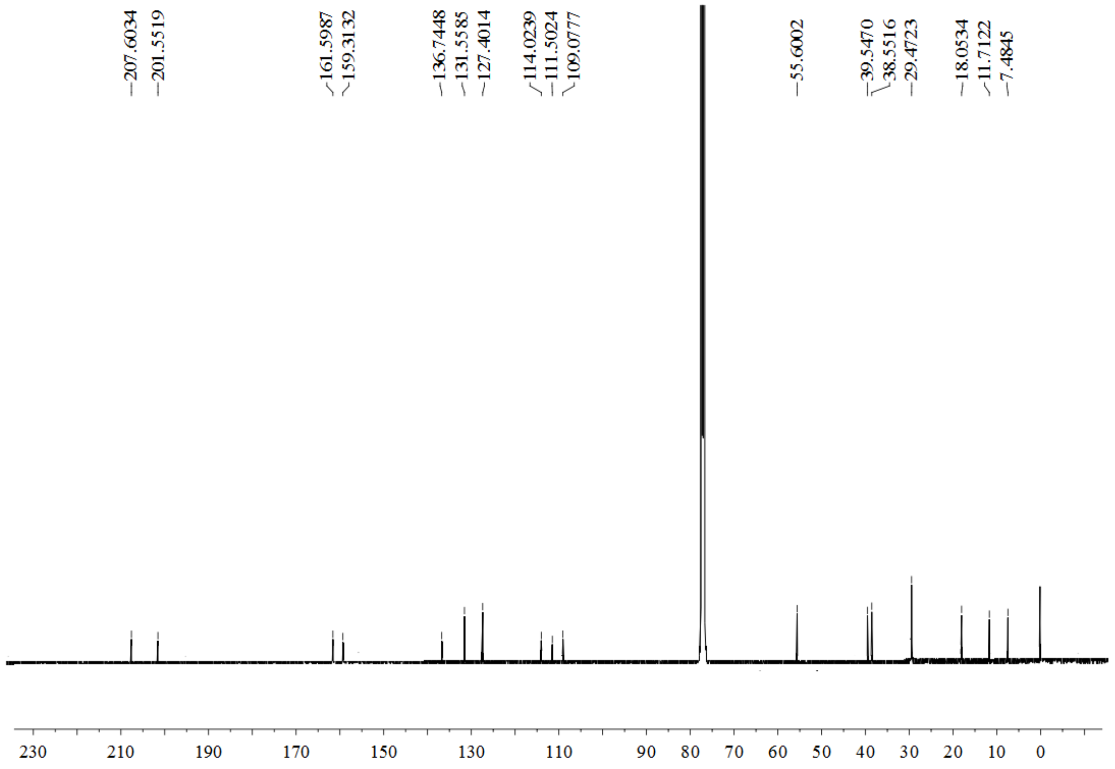

Figure S2. ^{13}C NMR spectra of sohiracillinone (**1**) in $CDCl_3$

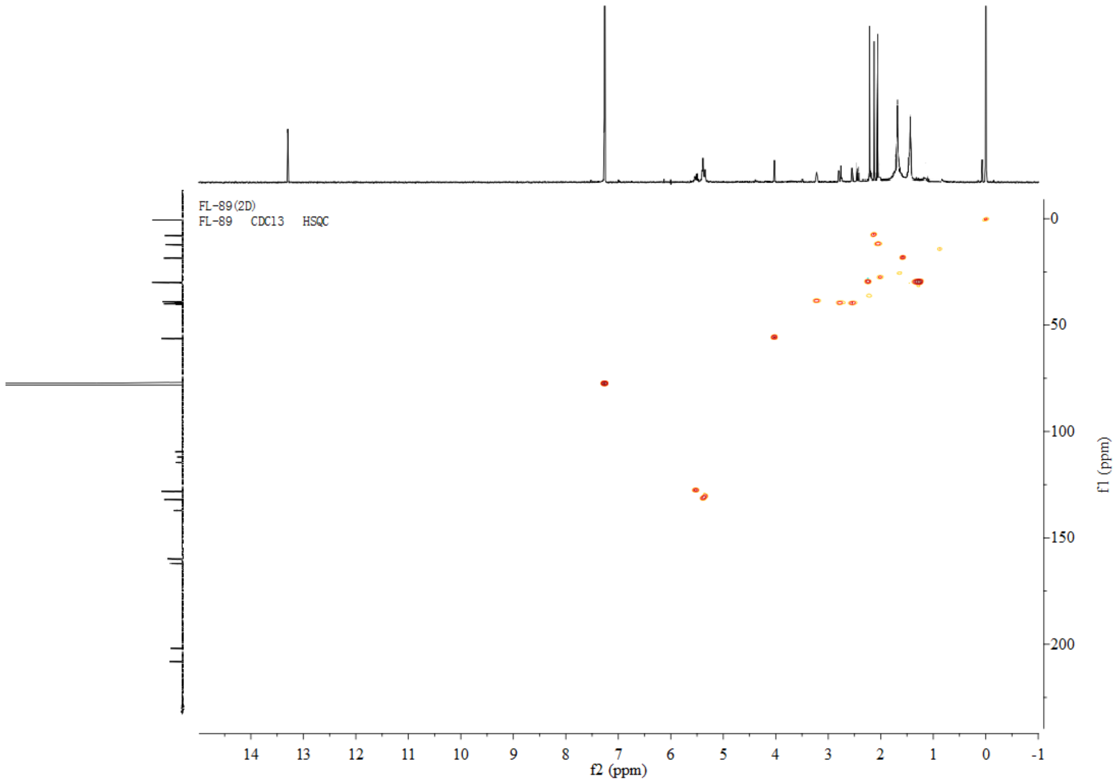

Figure S3. HSQC spectrum of sohiracillinone (**1**) in $CDCl_3$

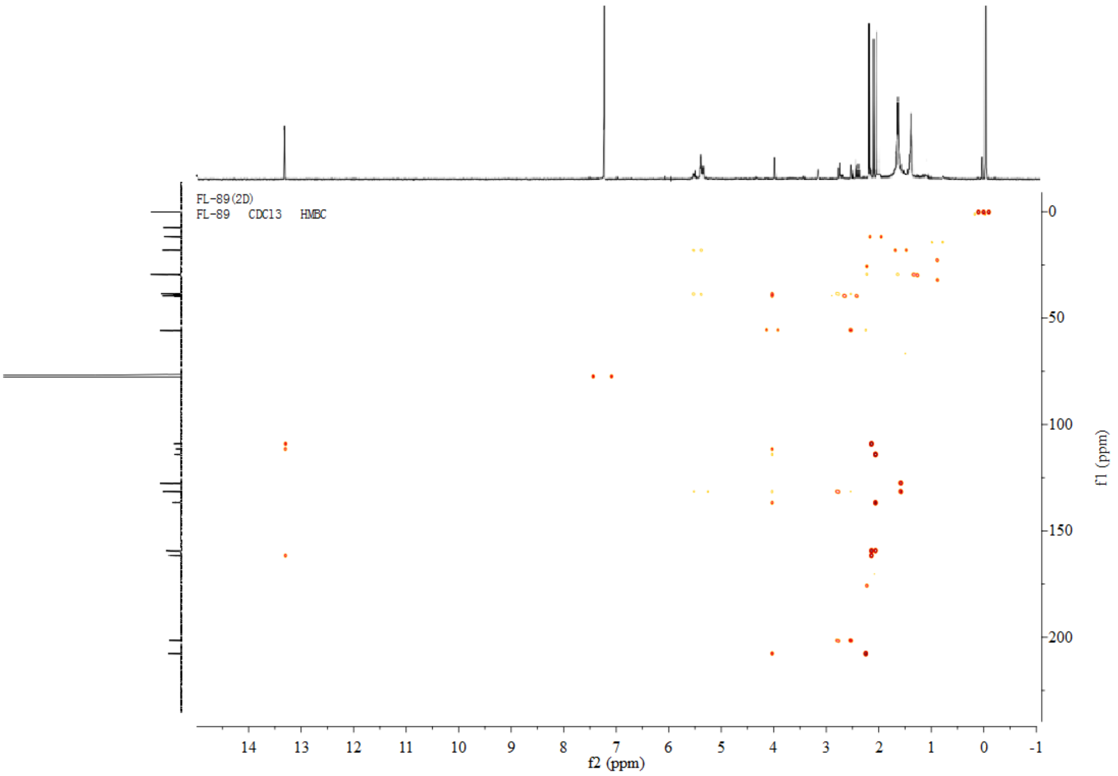

Figure S4. HMBC spectrum of sohiracillinone (**1**) in $CDCl_3$

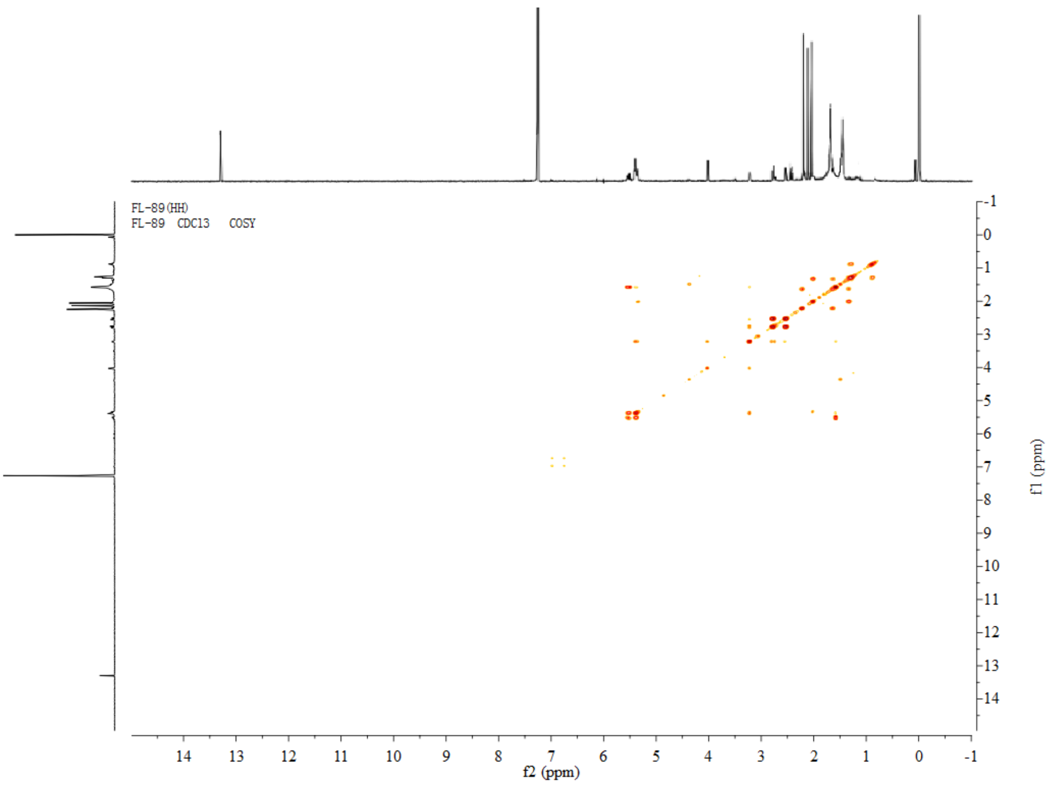

Figure S5. ^{1}H-^{1}H COSY spectrum of sohiracillinone (**1**) in $CDCl_3$

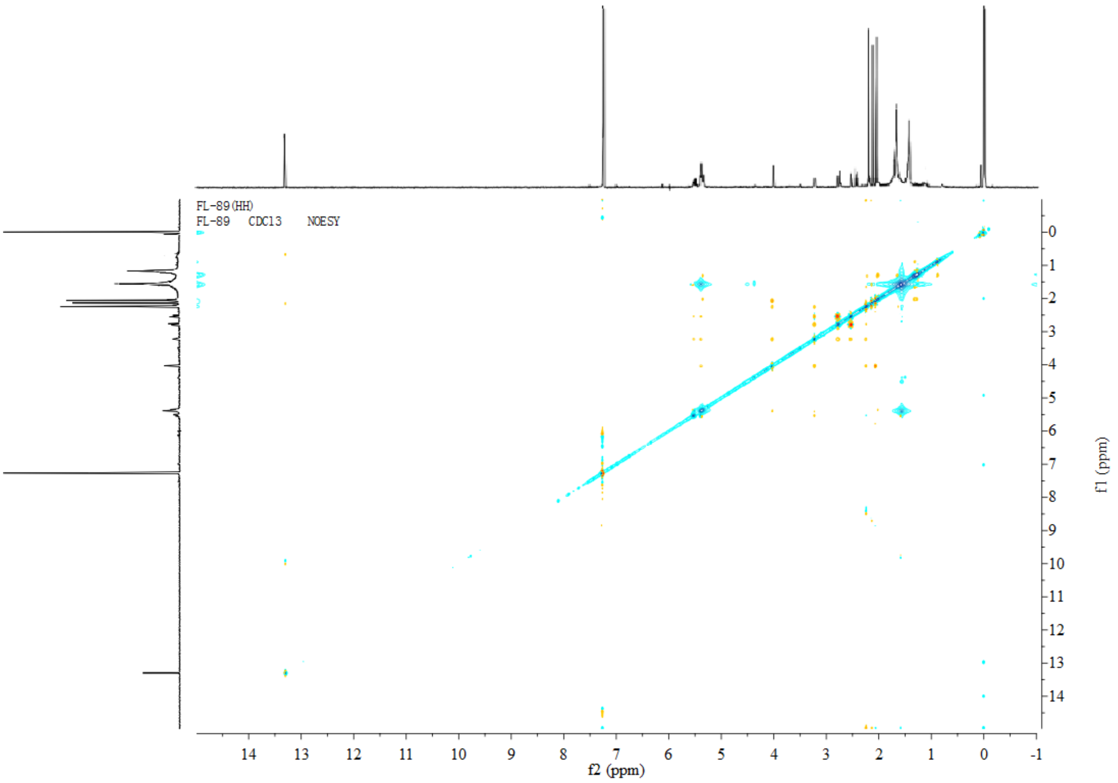

Figure S6. NOESY spectrum of sohiracillinone (**1**) in $CDCl_3$

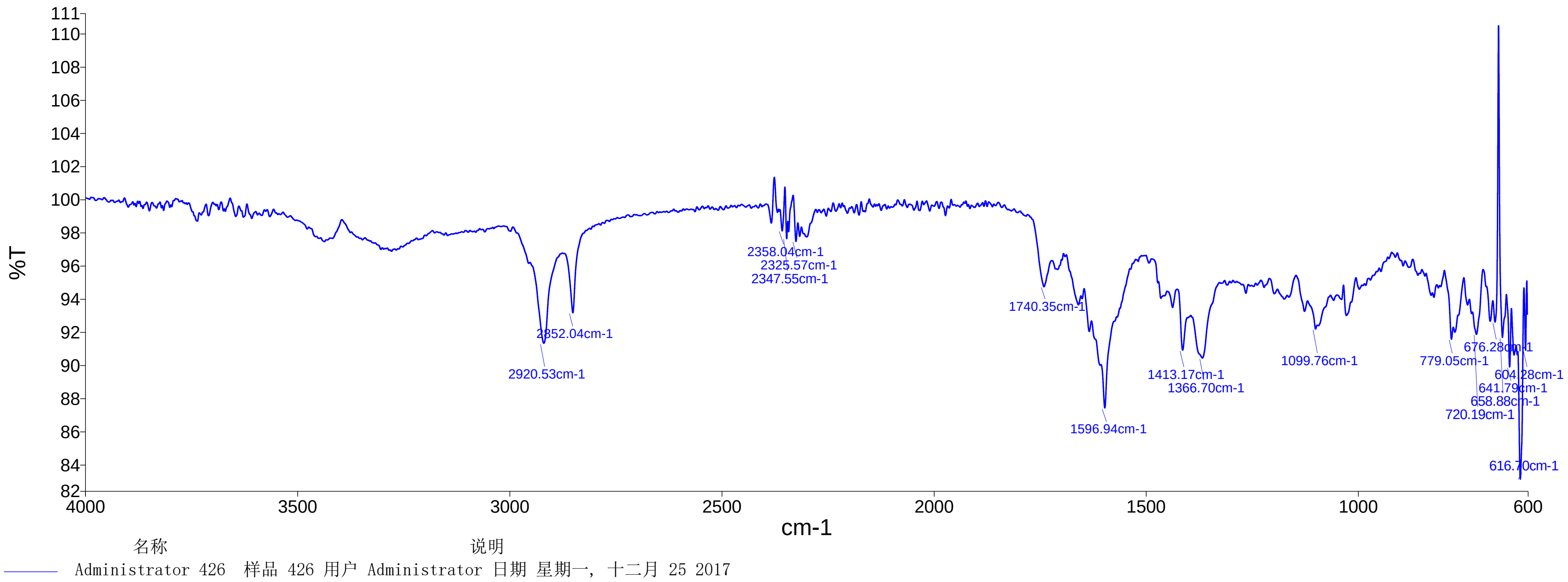

Figure S7. IR spectrum of sohiracillinone (**1**)

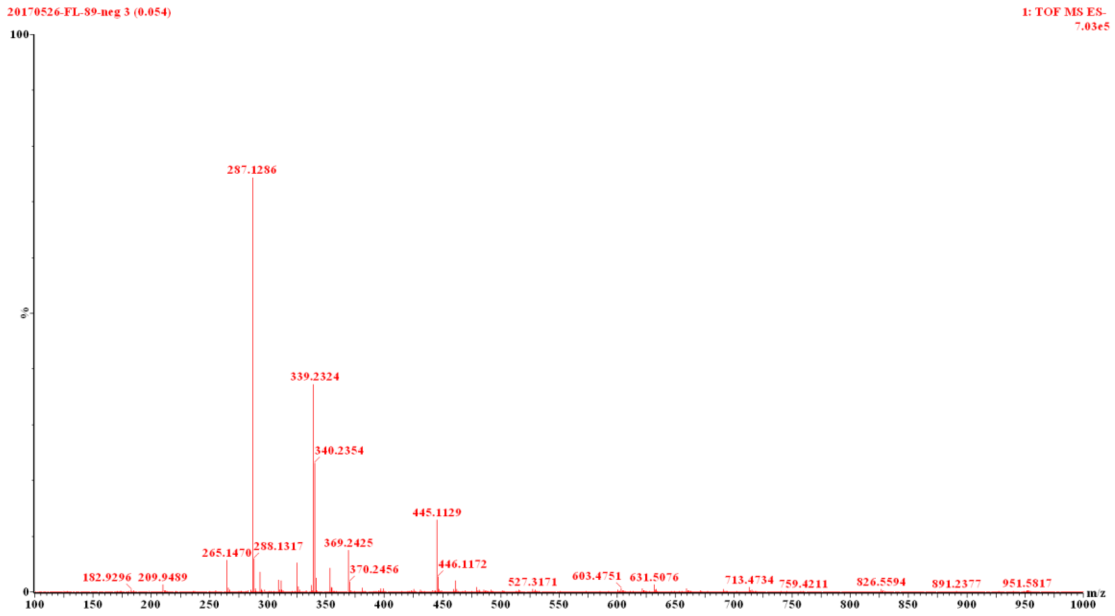

Figure S8. HRESIMS spectrum of sohiracillinone (**1**)

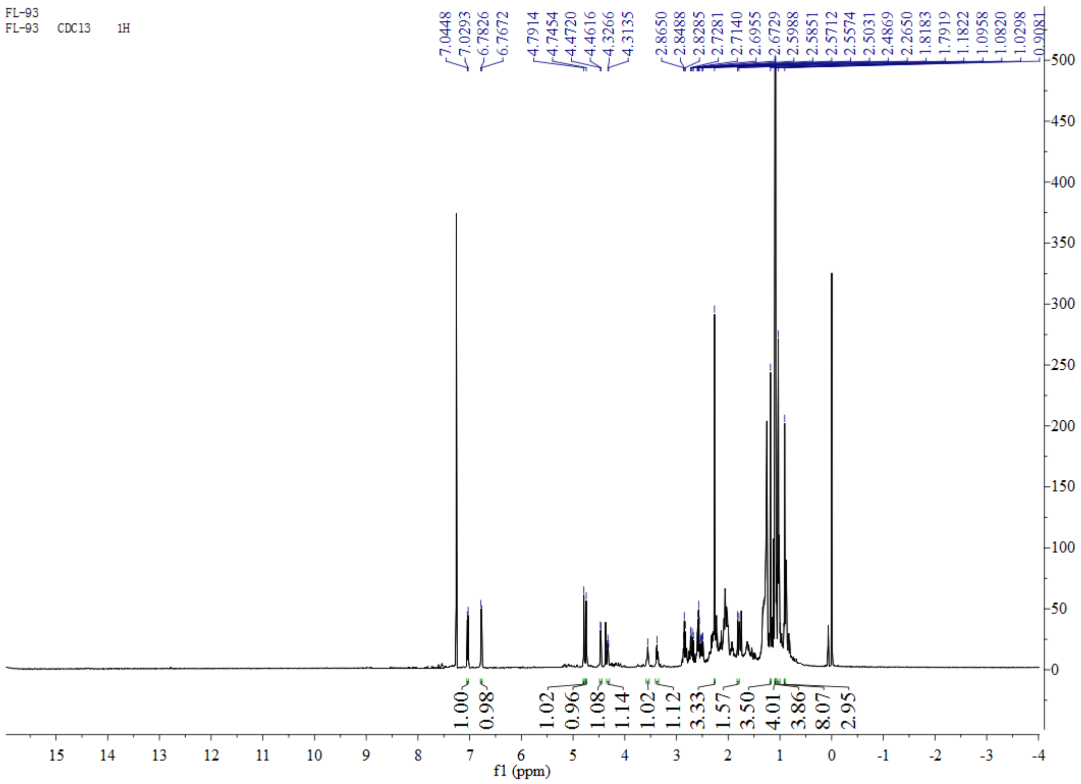

Figure S9. ¹H NMR spectrum of 11β-ethoxy-daedaleanic acid A (**2**) in $CDCl_3$

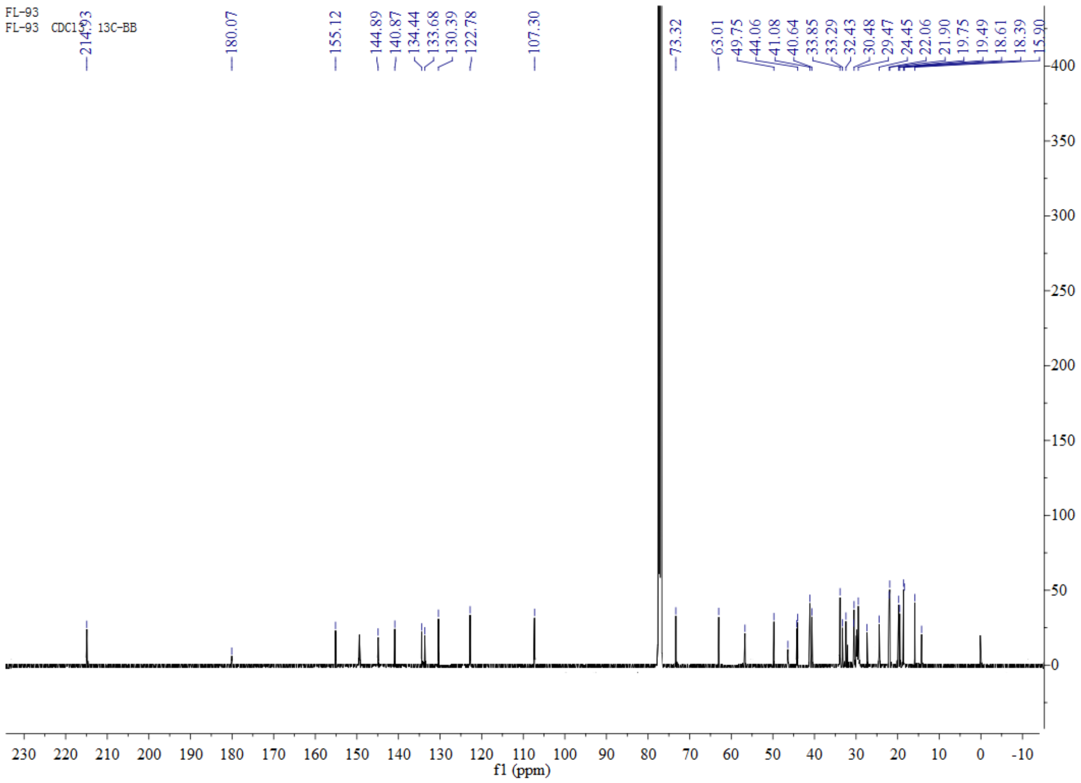

Figure S10. ¹³C NMR spectra of 11β-ethoxy-daedaleanic acid A (**2**) in $CDCl_3$

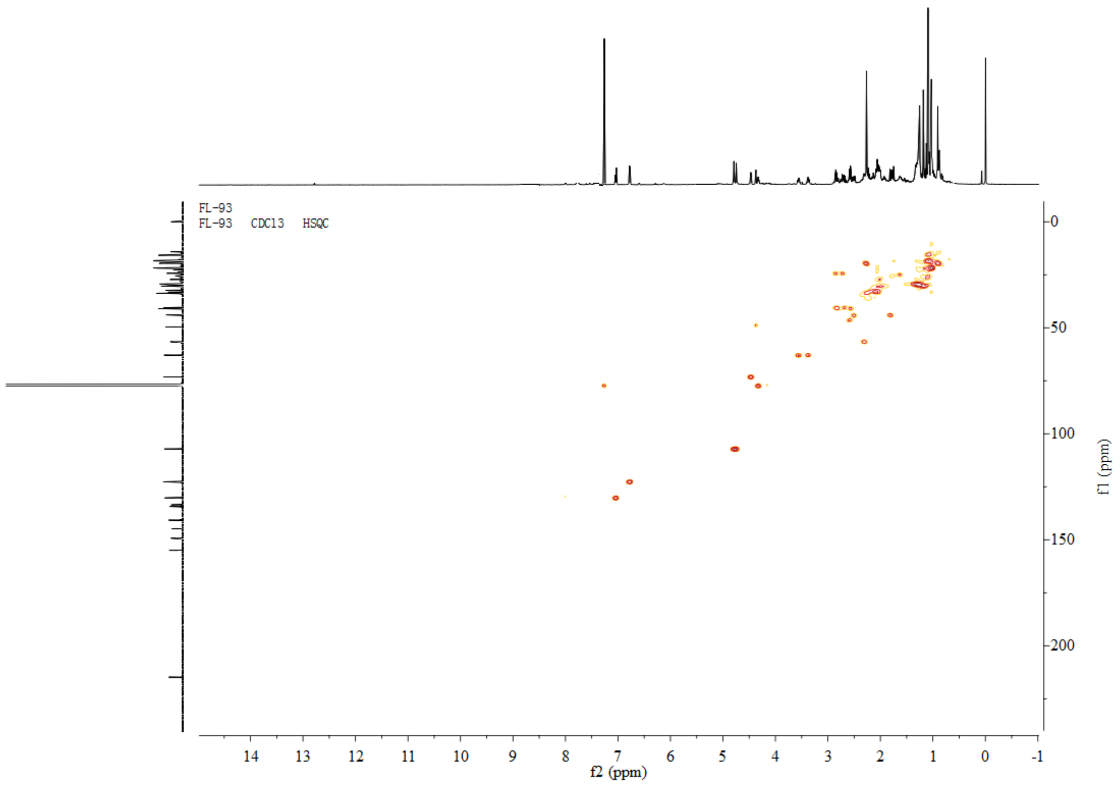

Figure S11. HSQC spectrum of 11*β*-ethoxy-daedaleanic acid A (**2**) in $CDCl_3$

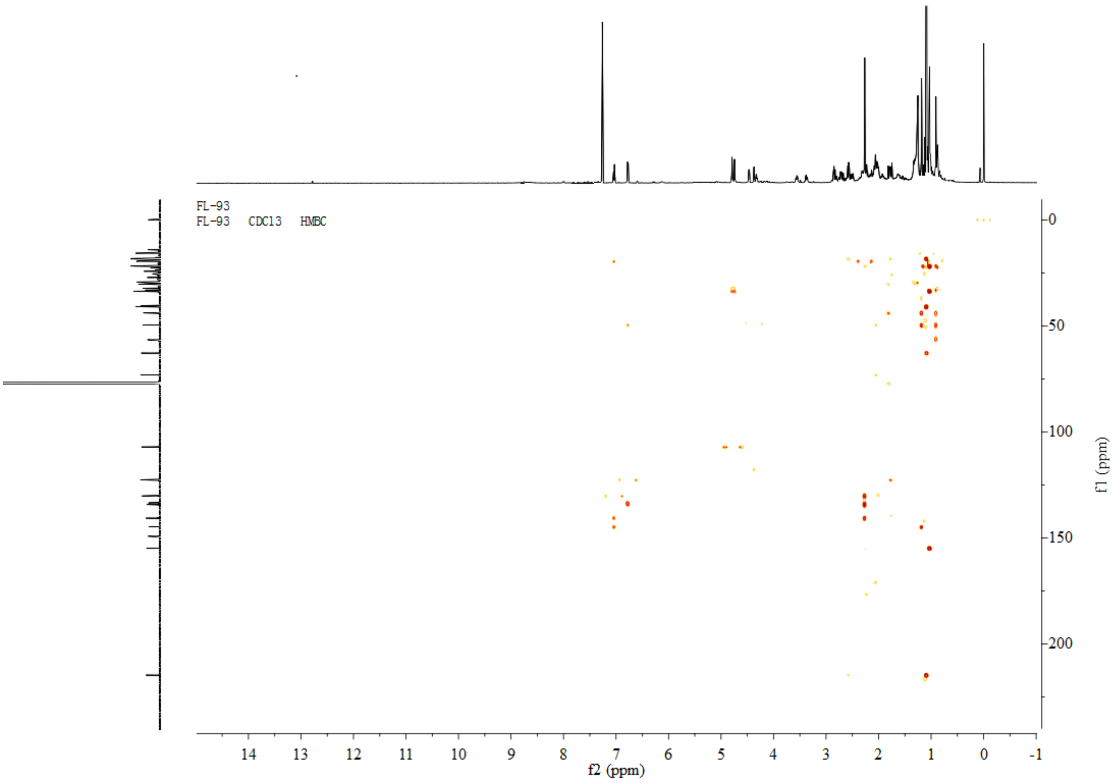

Figure S12. HMBC spectrum of 11*β*-ethoxy-daedaleanic acid A (**2**) in $CDCl_3$

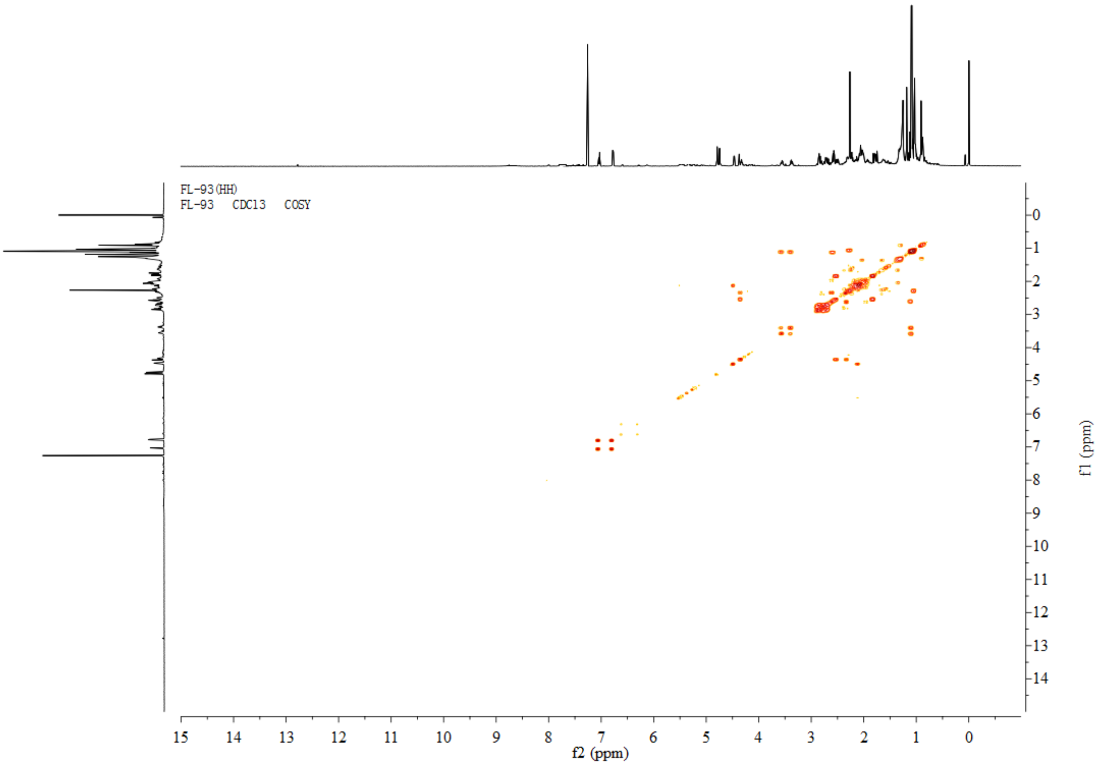

Figure S13. ^{1}H-^{1}H COSY spectrum of 11*β*-ethoxy-daedaleanic acid A (**2**) in $CDCl_3$

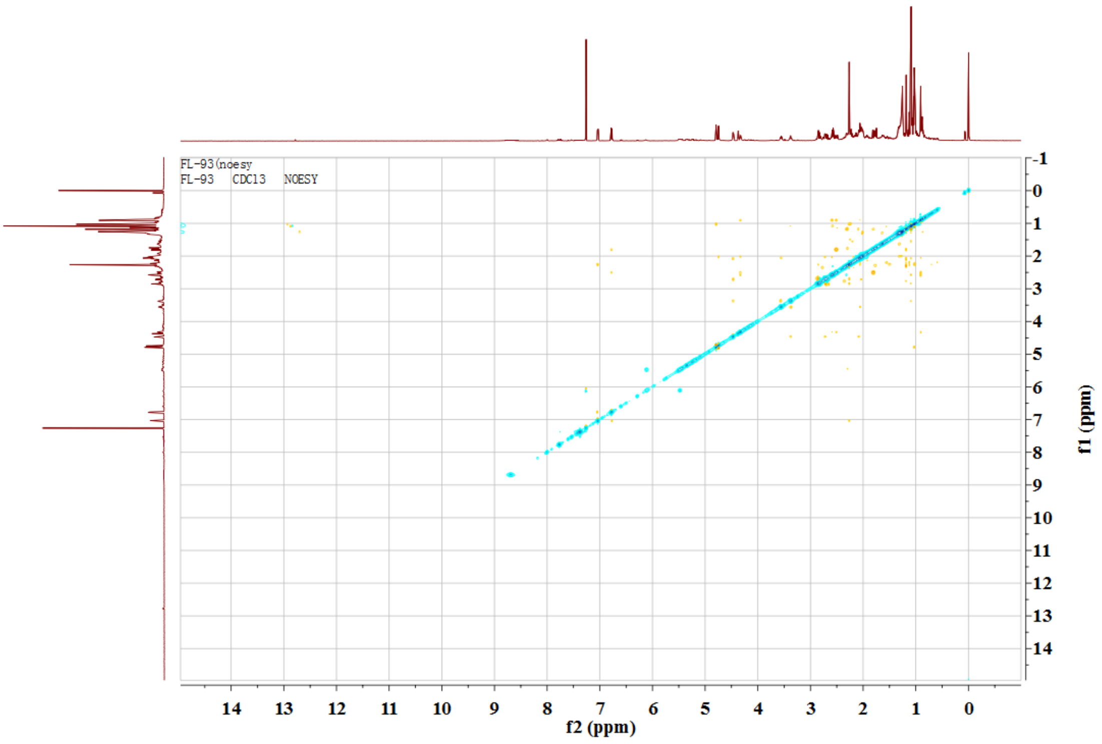

Figure S14. NOESY spectrum of 11*β*-ethoxy-daedaleanic acid A (**2**) in $CDCl_3$

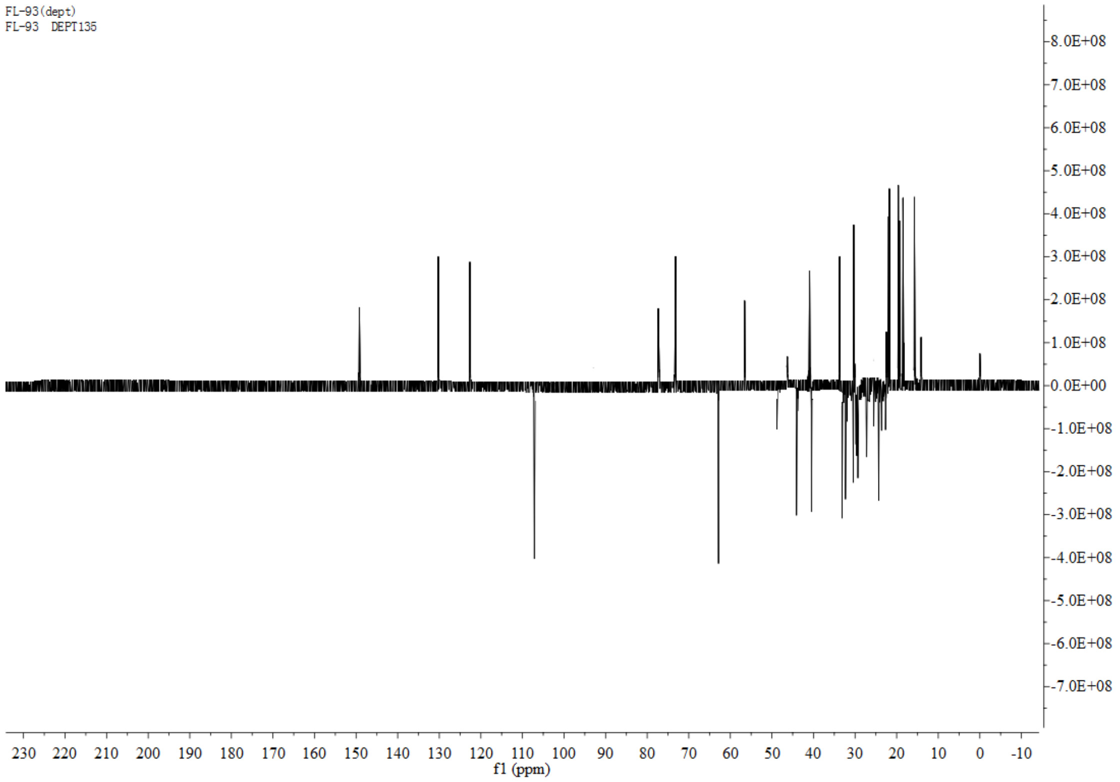

Figure S15. Dept spectrum of 11*β*-ethoxy-daedaleanic acid A (**2**) in $CDCl_3$

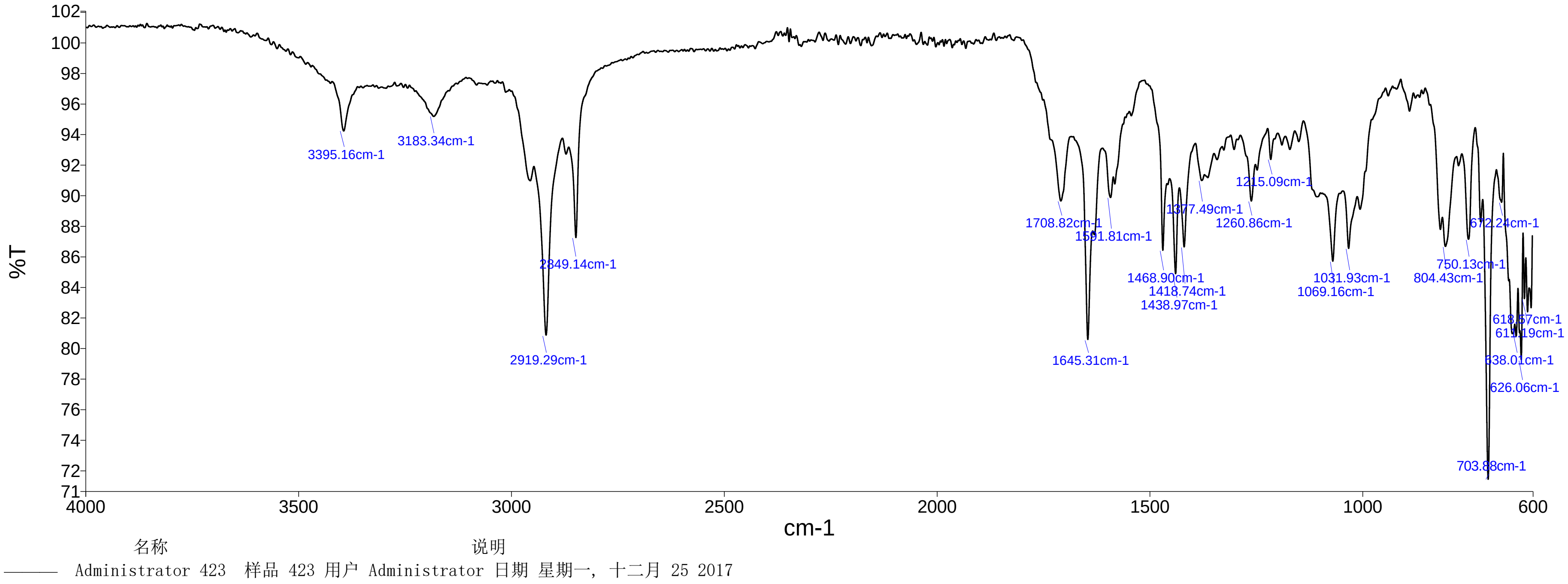

Figure S16. IR spectrum of 11β-ethoxy-daedaleanic acid A (**2**)

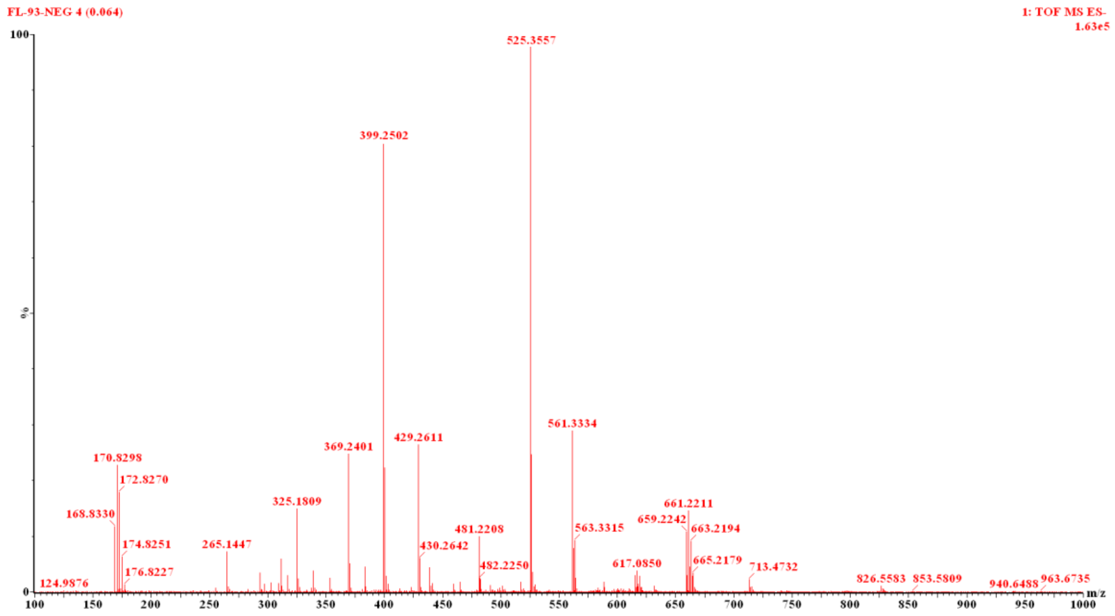

Figure S17. HRESIMS spectrum of 11β-ethoxy-daedaleanic acid A (**2**)

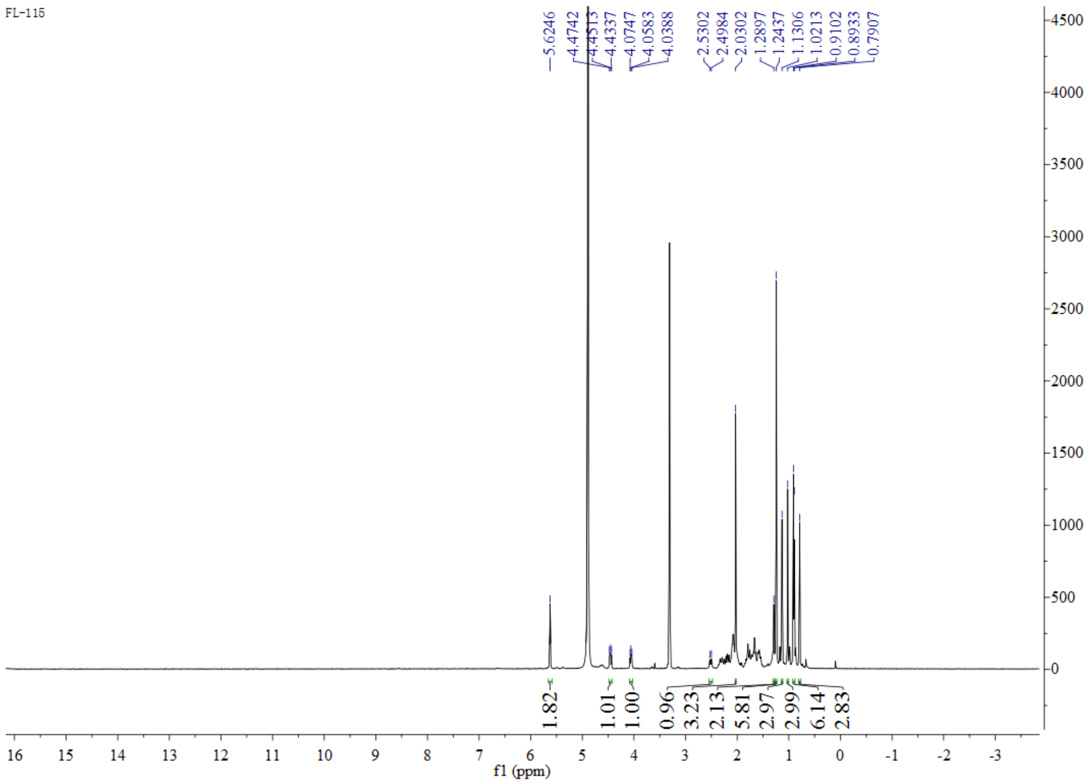

Figure S18. ^{1}H NMR spectrum of ceanphytamic acid A (**3**) in CD_3OD

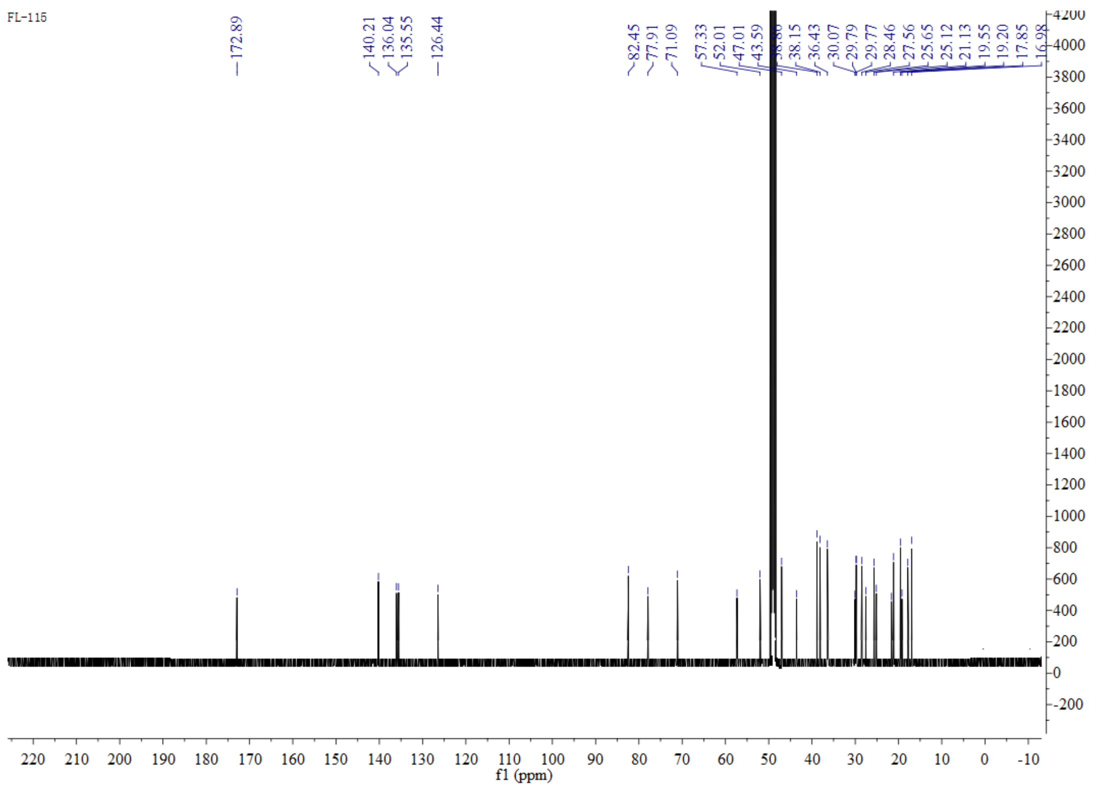

Figure S19. ^{13}C NMR spectra of ceanphytamic acid A (**3**) in CD_3OD

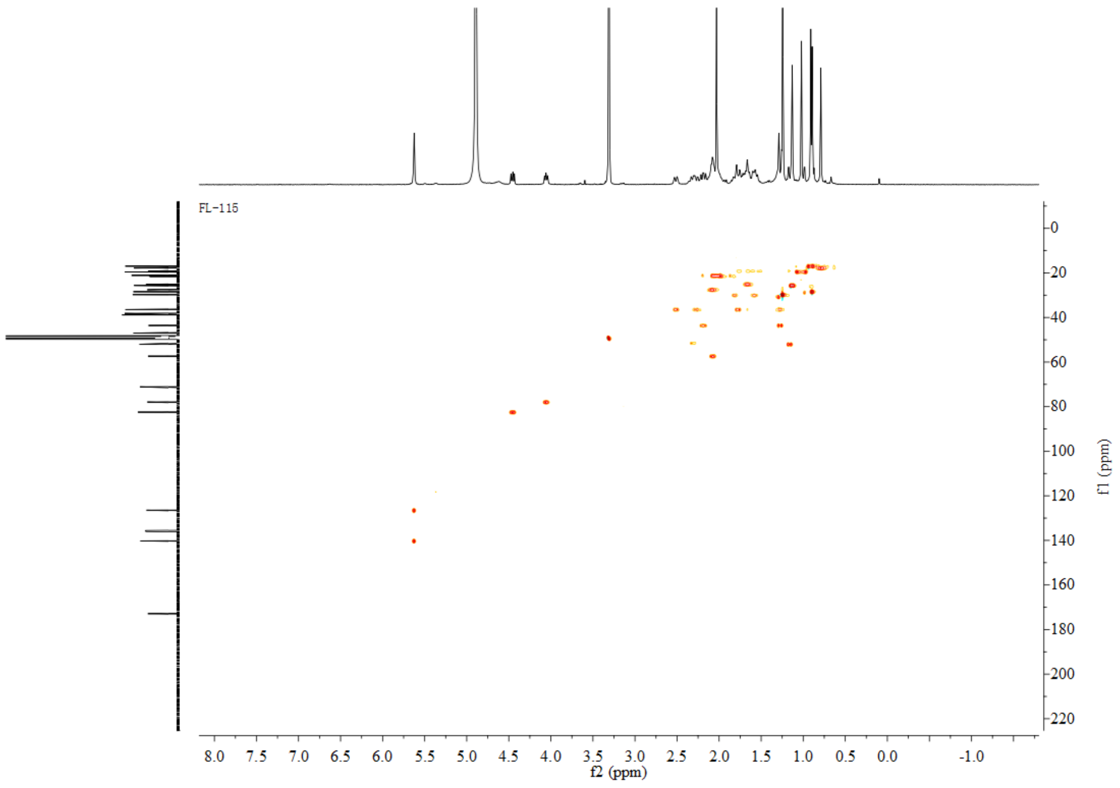

Figure S20. HSQC spectrum of ceanphytamic acid A (**3**) in CD_3OD

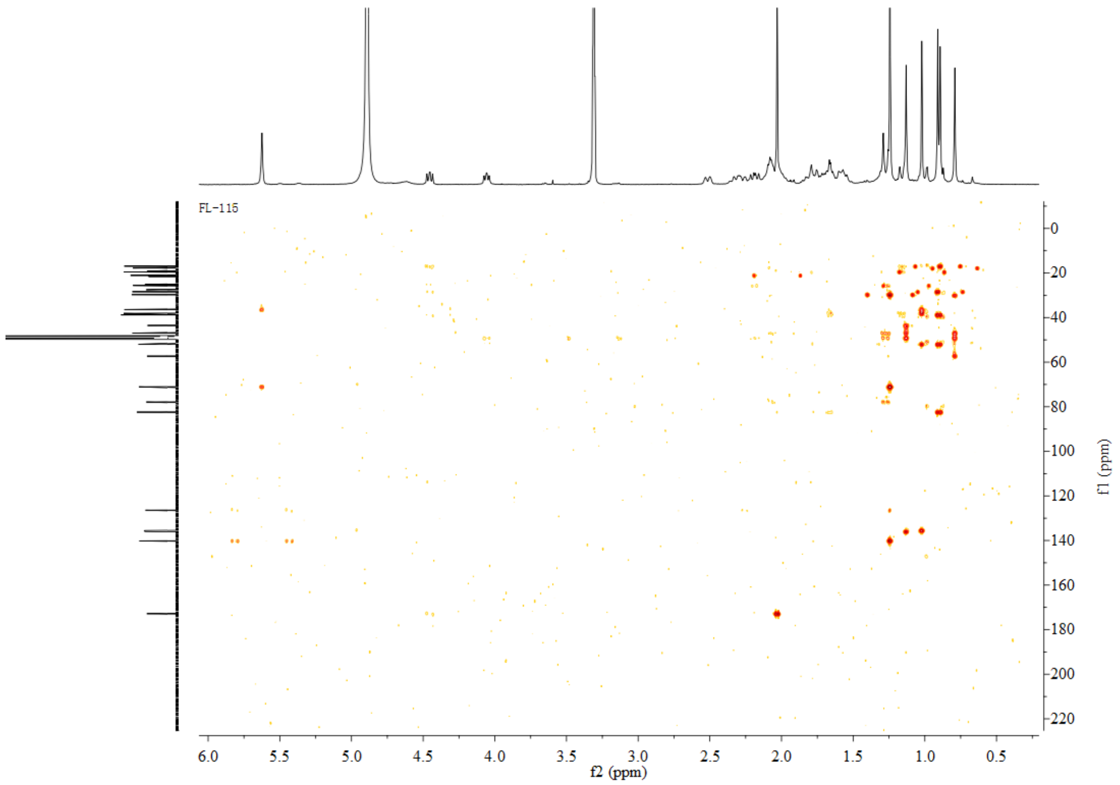

Figure S21. HMBC spectrum of ceanphytamic acid A (**3**) in CD_3OD

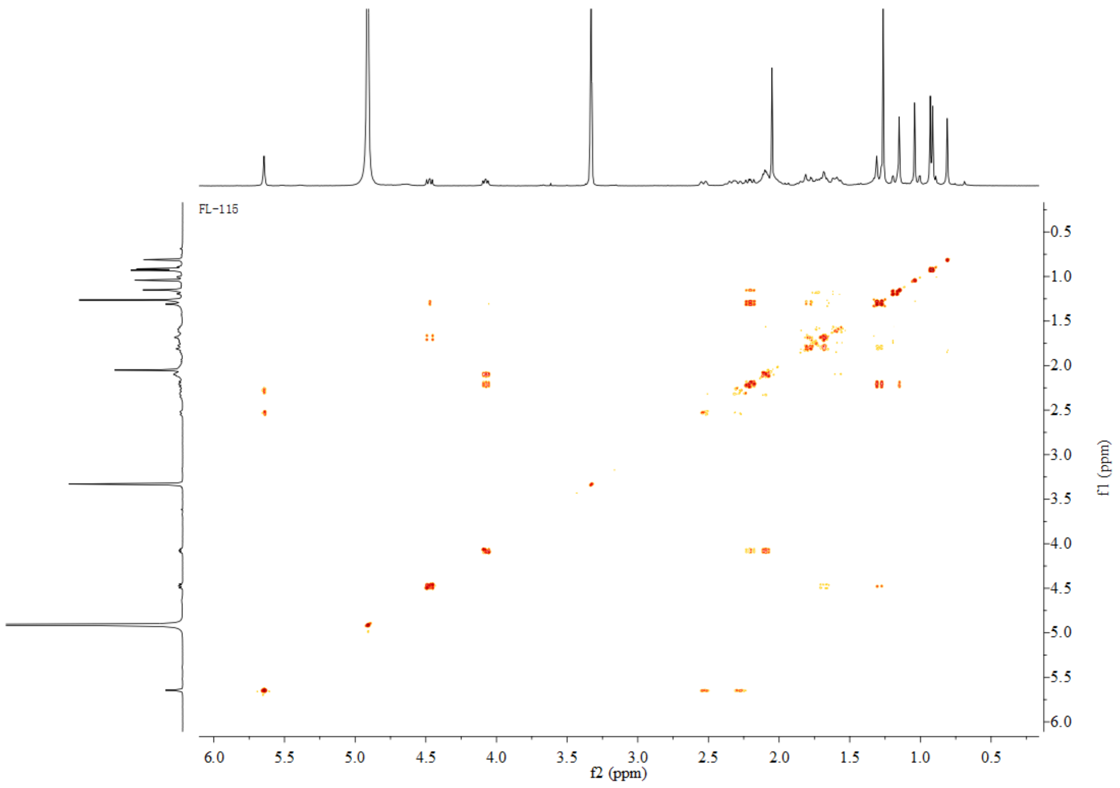

Figure S22. ^{1}H-^{1}H COSY spectrum of ceanphytamic acid A (**3**) in CD_3OD

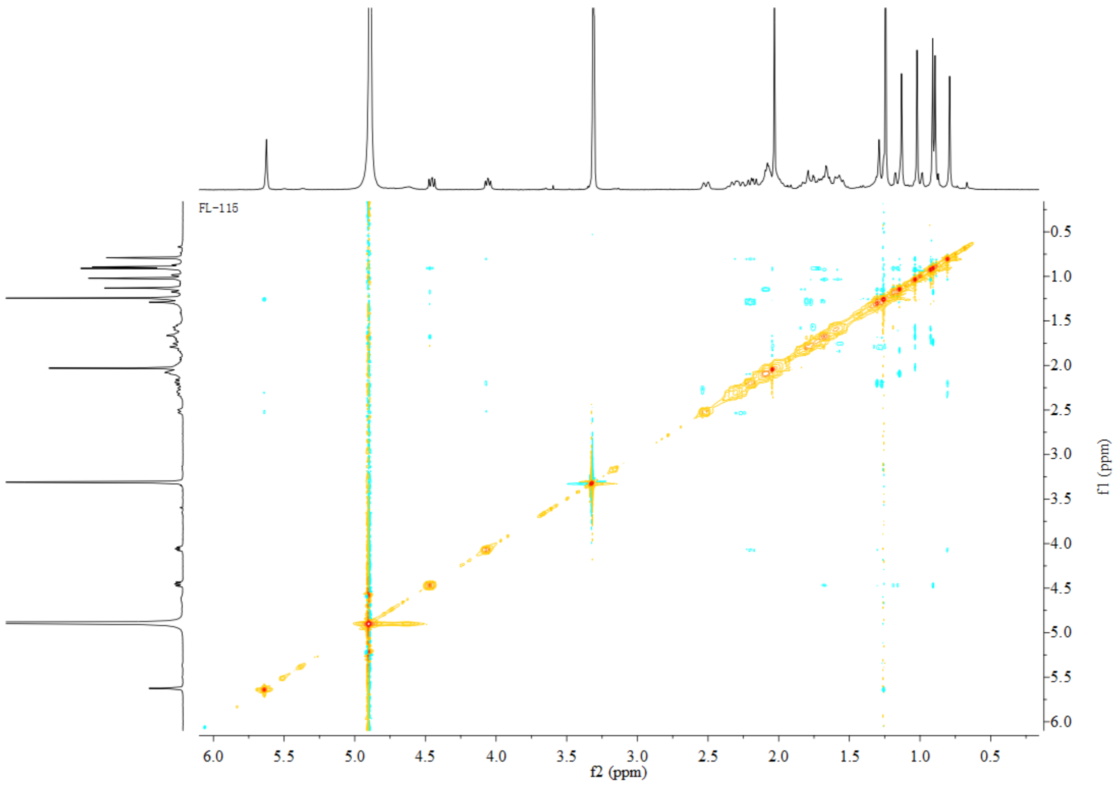

Figure S23. NOESY spectrum of ceanphytamic acid A (**3**) in CD_3OD

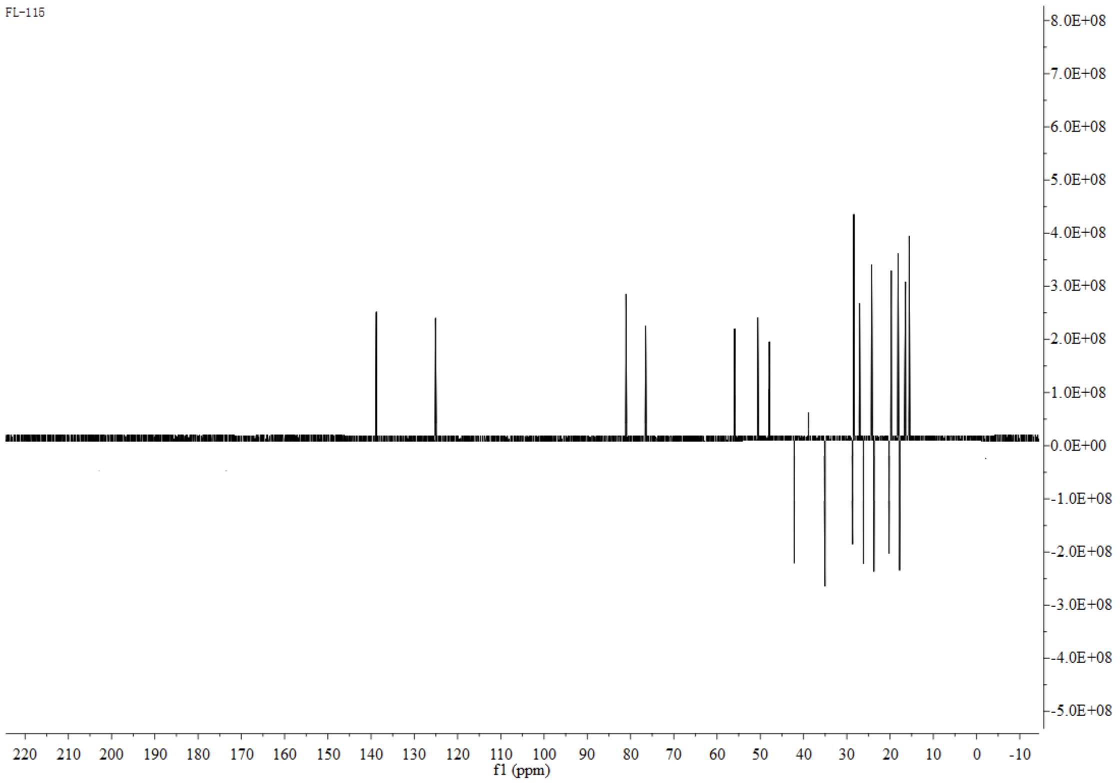

Figure S24. Dept spectrum of ceanphytamic acid A (**3**) in CD_3OD

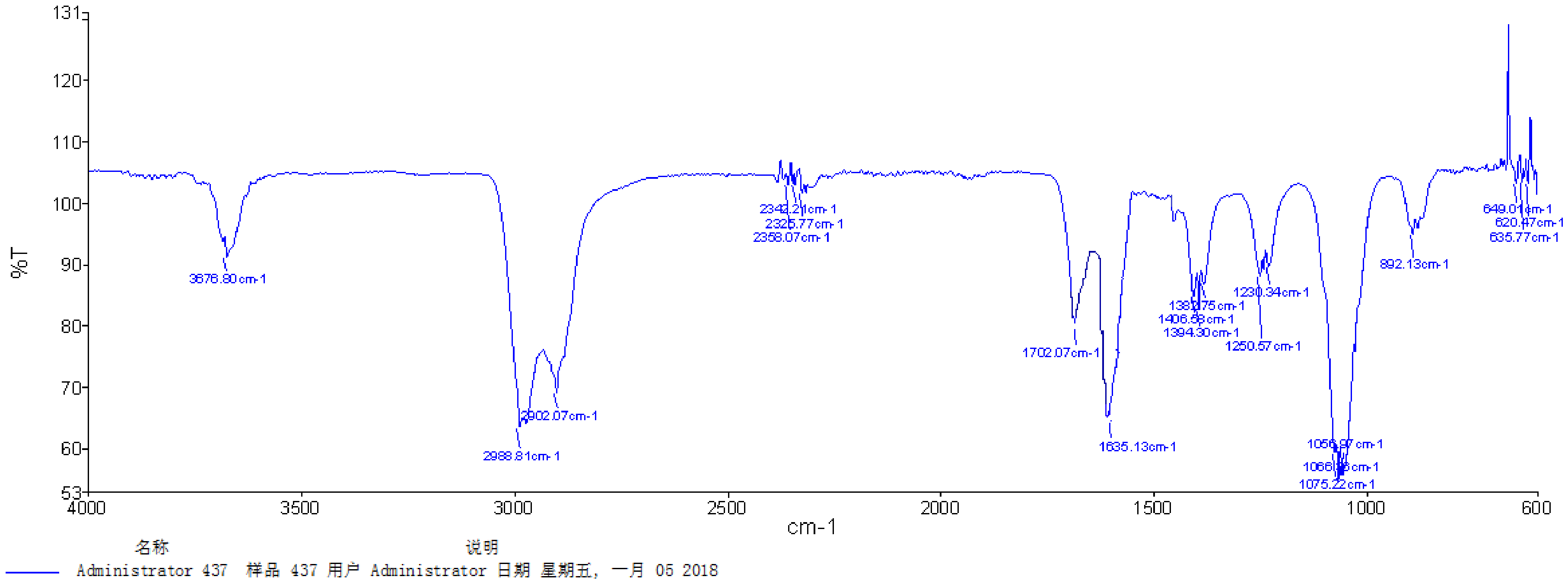

Figure S25. IR spectrum of ceanphytamic acid A (**3**)

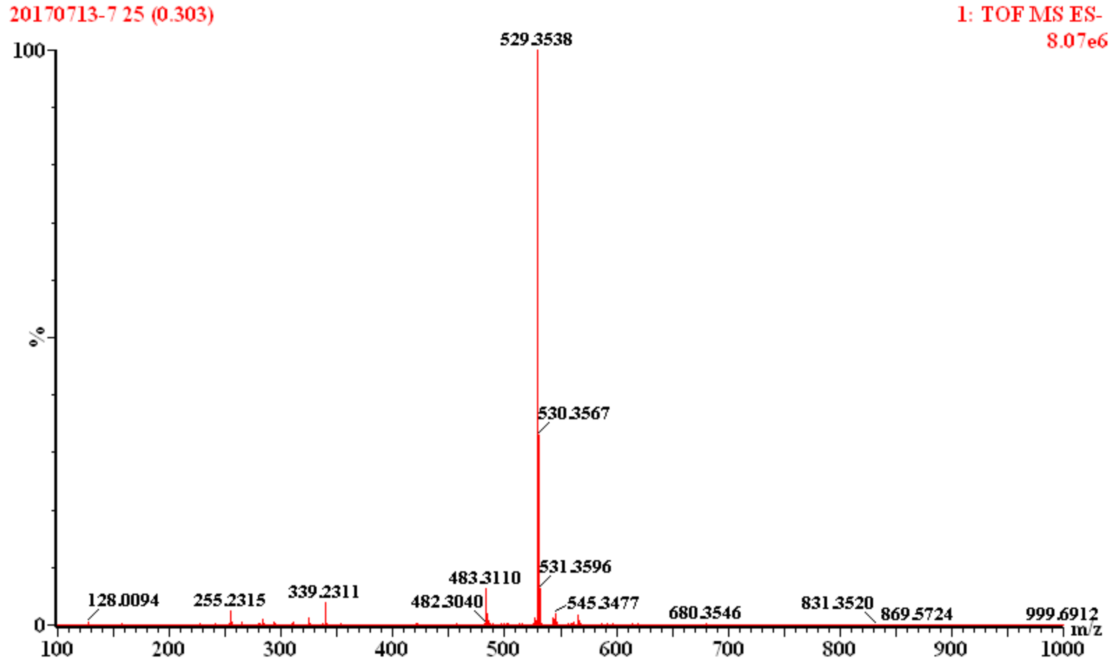

Figure S26. HRESIMS spectrum of ceanphytamic acid A (**3**)

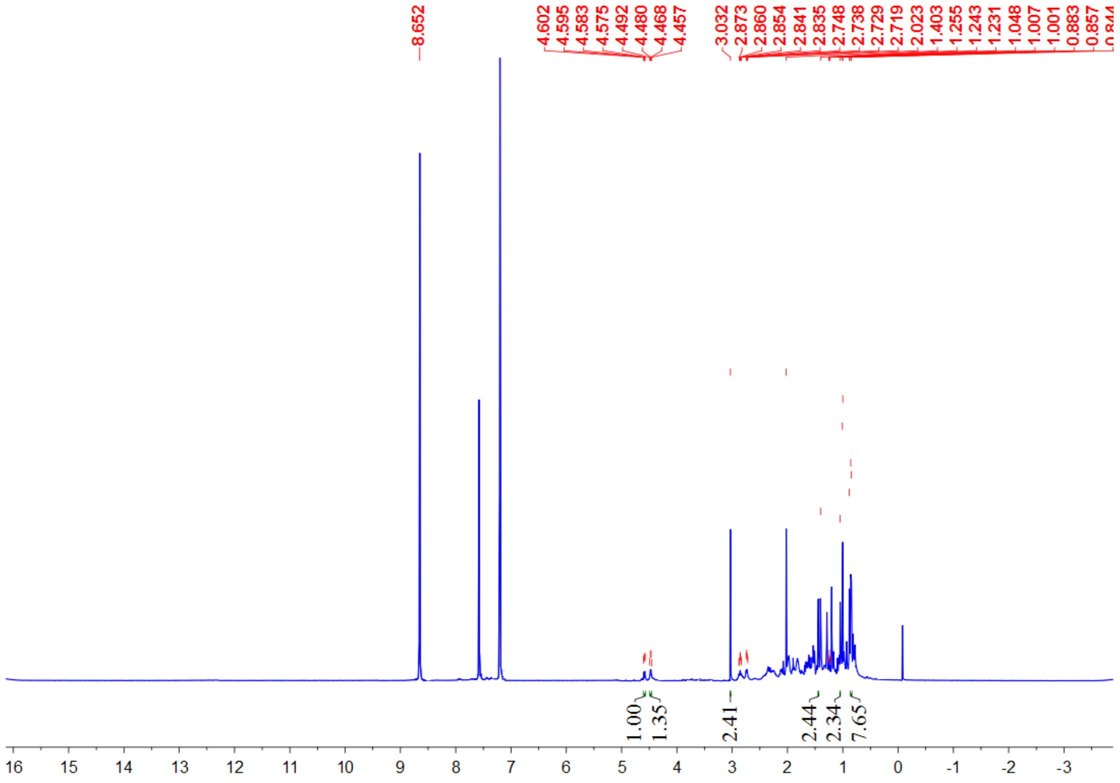

Figure S27. ^{1}H NMR spectrum of ceanphytamic acid B (**4**) in C_5D_5N-*d5*

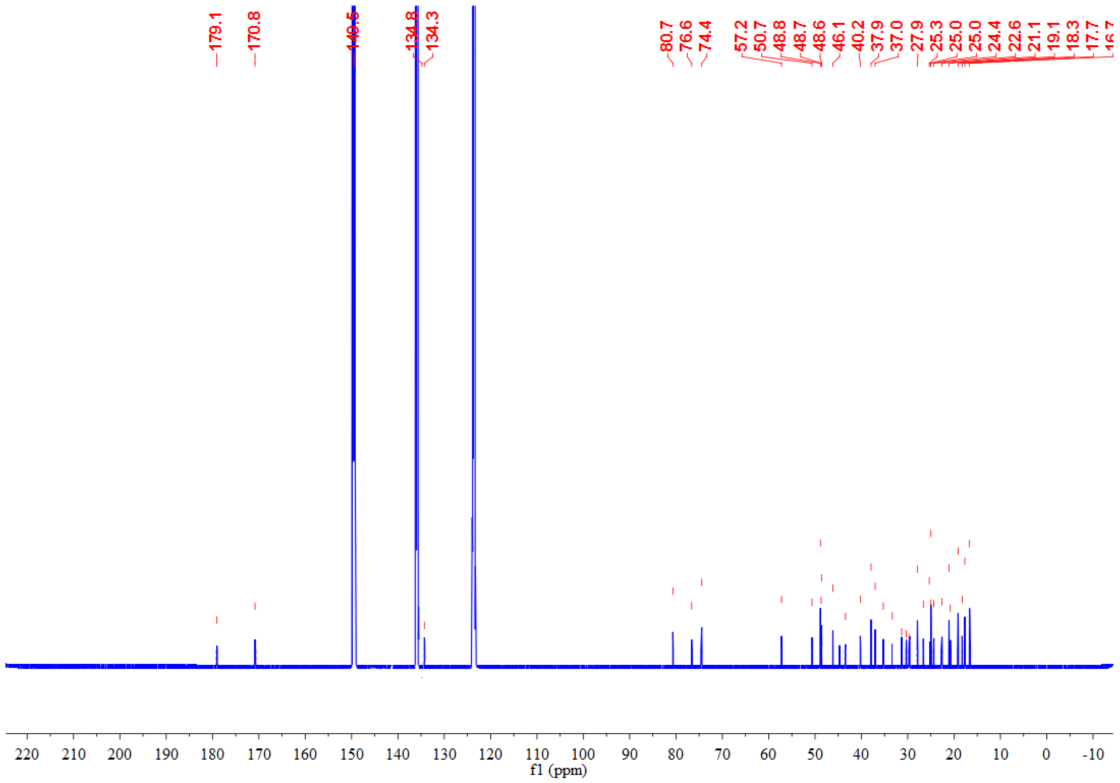

Figure S28. ^{13}C NMR spectra of ceanphytamic acid B (**4**) in C_5D_5N-*d5*

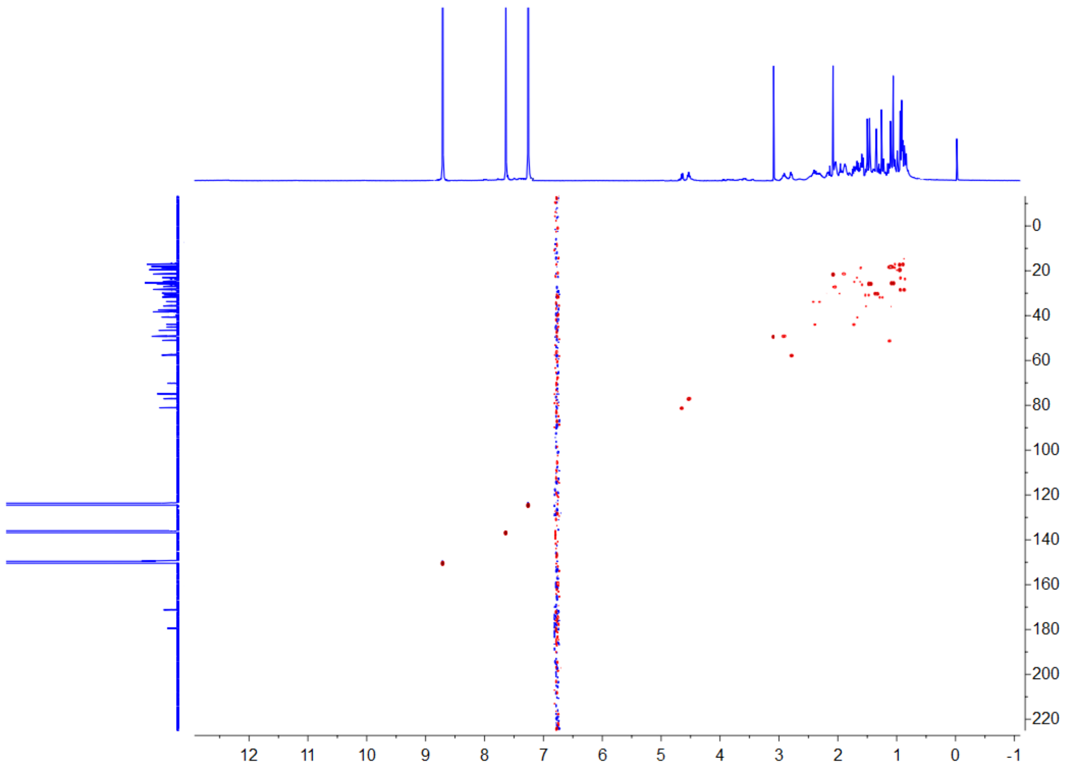

Figure S29. HSQC spectrum of ceanphytamic acid B (**4**) in C_5D_5N-*d5*

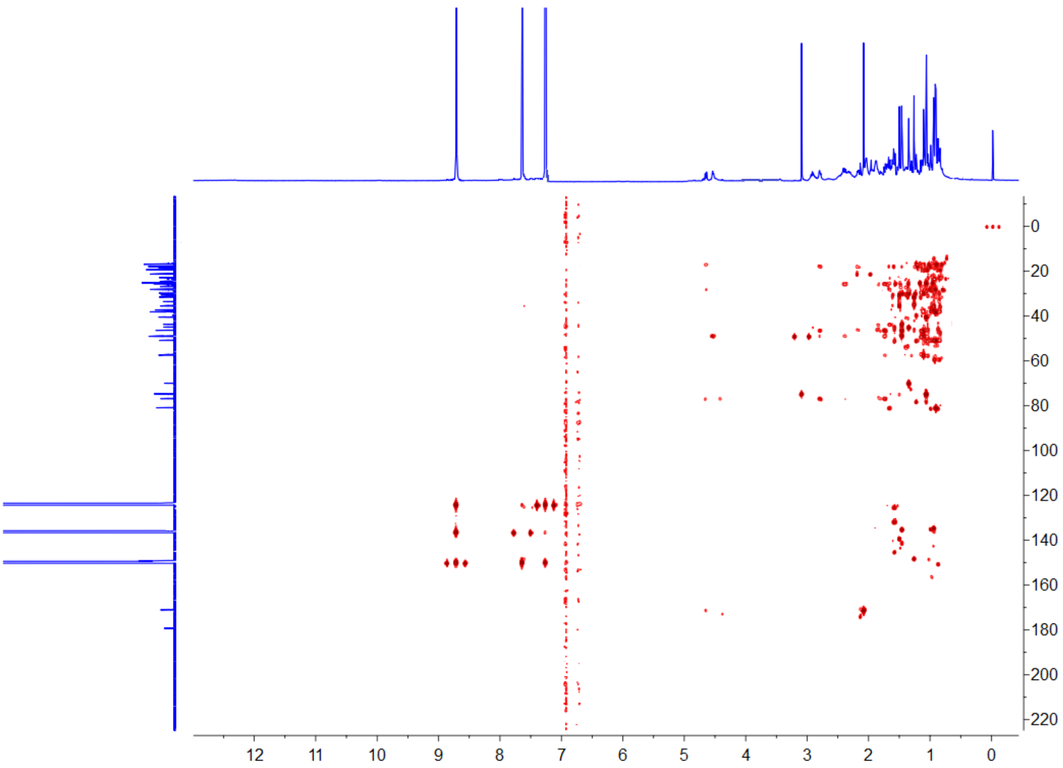

Figure S30. HMBC spectrum of ceanphytamic acid B (**4**) in C_5D_5N-*d5*

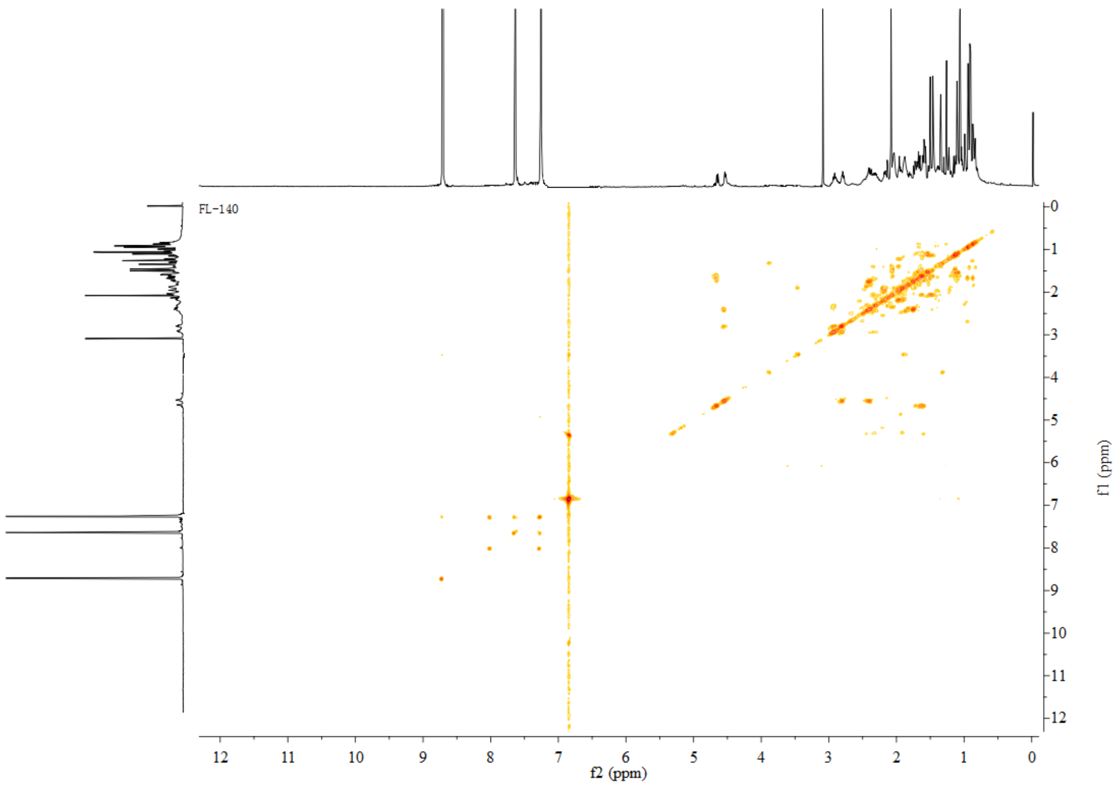

Figure S31. ^{1}H-^{1}H COSY spectrum of ceanphytamic acid B (**4**) in C_5D_5N-*d5*

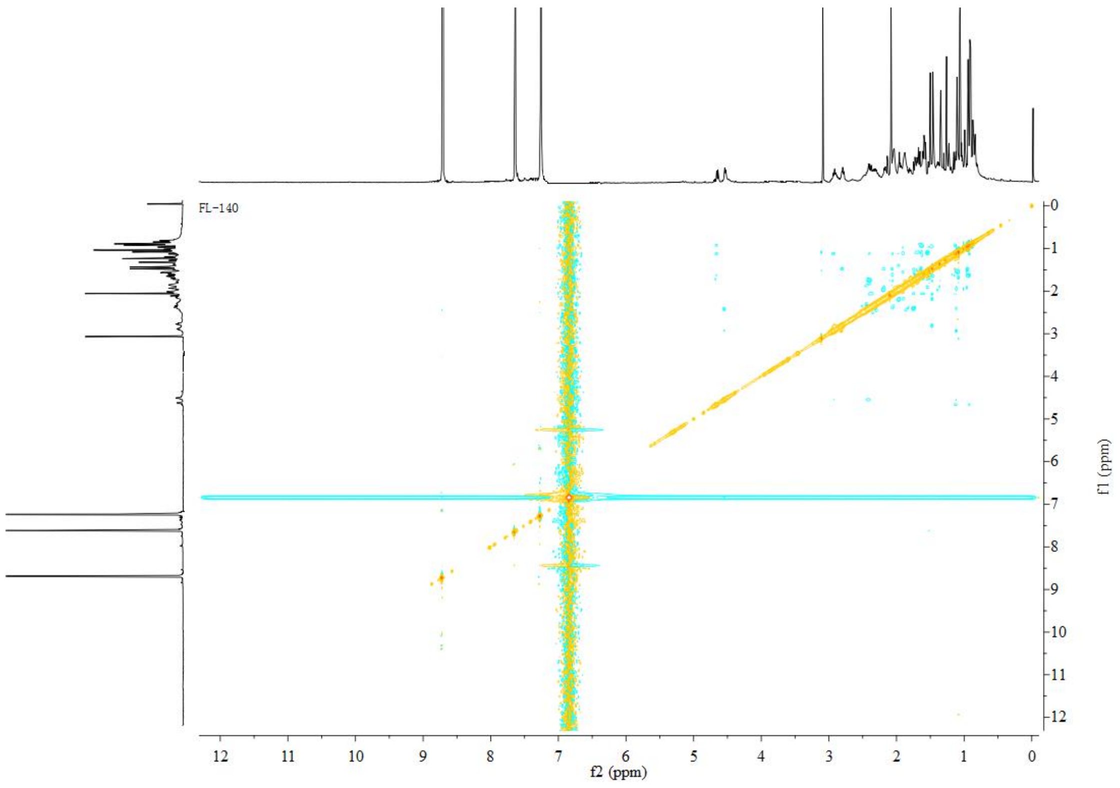

Figure S32. NOESY spectrum of ceanphytamic acid B (**4**) in C_5D_5N-*d5*

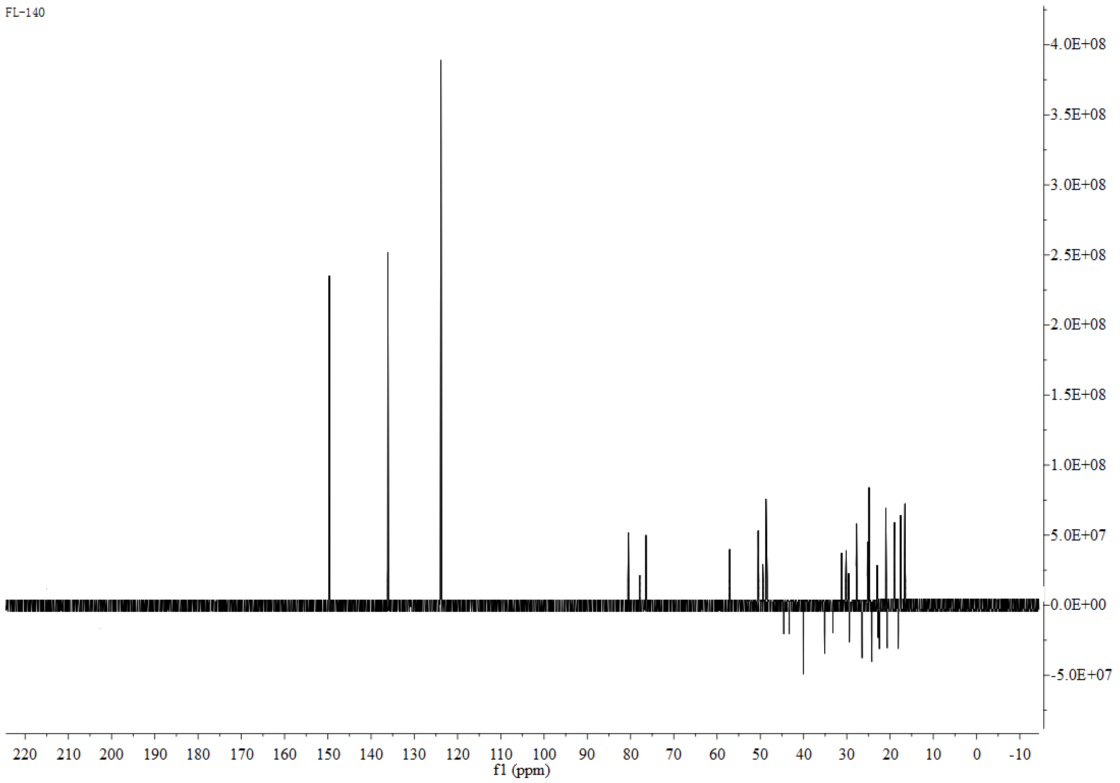

Figure S33. Dept spectrum of ceanphytamic acid B (**4**) in C_5D_5N-*d5*

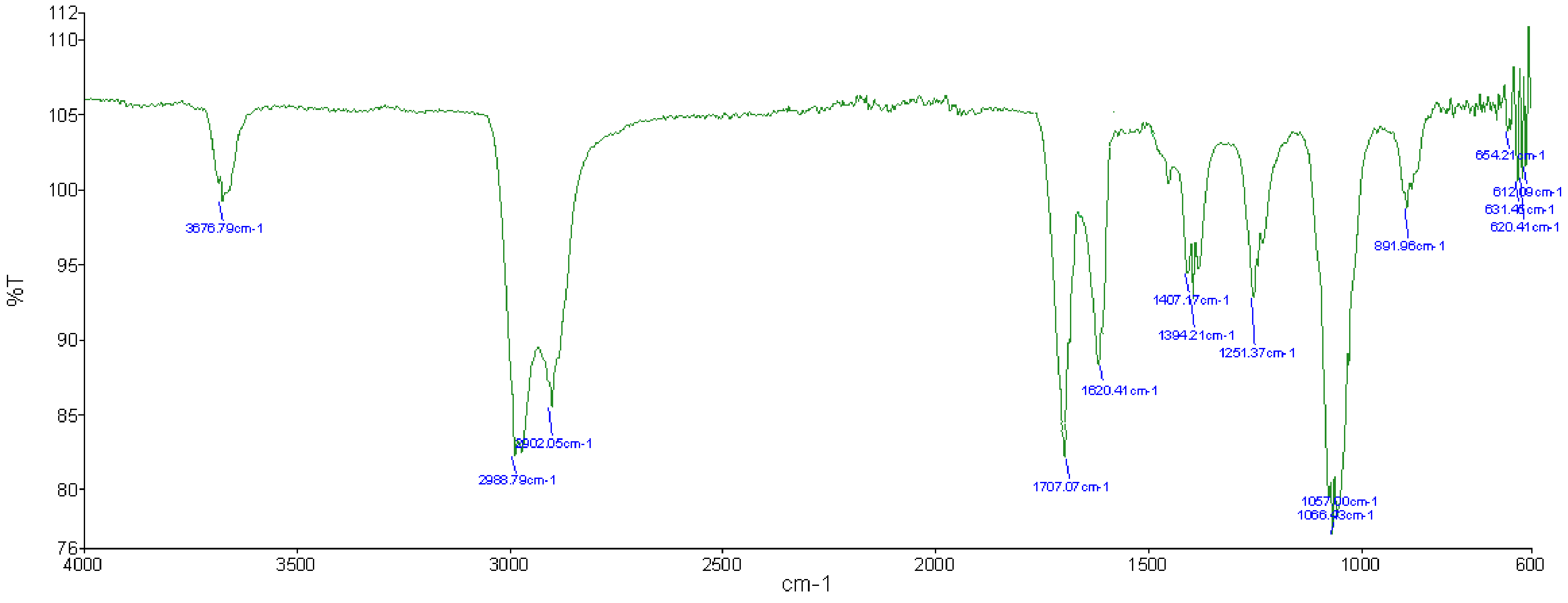

Figure S34. IR spectrum of ceanphytamic acid B (**4**)

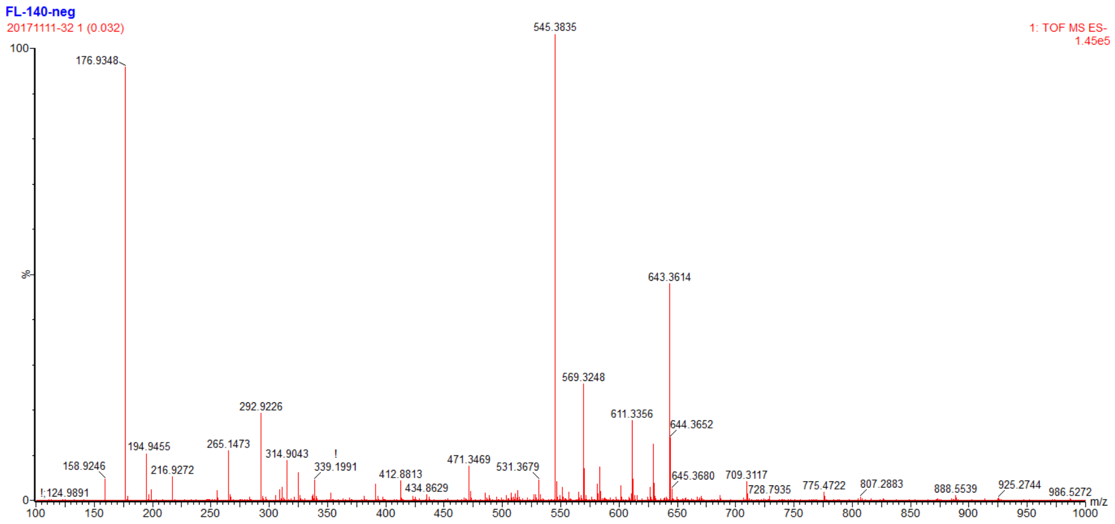

Figure S35. HRESIMS spectrum of ceanphytamic acid B (**4**)
